# Patient‐Derived Organoids Can Guide Personalized‐Therapies for Patients with Advanced Breast Cancer

**DOI:** 10.1002/advs.202101176

**Published:** 2021-10-04

**Authors:** Ping Chen, Xu Zhang, Renbo Ding, Linglin Yang, Xueying Lyu, Jianming Zeng, Josh Haipeng Lei, Lijian Wang, Jiong Bi, Nan Shao, Ditian Shu, Bin Wu, Jingbo Wu, Zhihui Yang, Haiyan Wang, Biqiong Wang, Kang Xiong, Yun Lu, Shaozhi Fu, Tak Kan Choi, Ng Wai Lon, Aiping Zhang, Dongyang Tang, Yingyao Quan, Ya Meng, Kai Miao, Heng Sun, Ming Zhao, Jiaolin Bao, Lei Zhang, Xiaoling Xu, Yanxia Shi, Ying Lin, Chuxia Deng

**Affiliations:** ^1^ Cancer Centre Faculty of Health Sciences University of Macau Macau SAR 999078 China; ^2^ Institute of Translational Medicine Faculty of Health Sciences University of Macau Macau SAR 999078 China; ^3^ MOE Frontier Science Centre for Precision Oncology University of Macau Macau SAR 999078 China; ^4^ Department of Oncology The Affiliated Hospital of Southwest Medical University Luzhou Sichuan 646000 China; ^5^ Laboratory of Surgery The First Affiliated Hospital Sun Yat‐Sen University No. 58 of Zhongshan 2nd road, Yuexiu district Guangzhou Guangdong 510080 China; ^6^ Breast Disease Center The First Affiliated Hospital Sun Yat‐Sen University No.58 of Zhongshan 2nd road, Yuexiu district Guangzhou Guangdong 510080 China; ^7^ Department of Medical Oncology Sun Yet‐sen University Cancer Center Collaborative Innovation Center of Cancer Medicine State Key Laboratory of Oncology in South China Sun Yat‐sen University Guangzhou Guangdong 510060 China; ^8^ Department of Breast Surgery The Affiliated Hospital of Southwest Medical University Luzhou Sichuan 646000 China; ^9^ Department of Pathology The Affiliated Hospital of Southwest Medical University Luzhou Sichuan 646000 China; ^10^ Centro Hospitalar Conde de S. Januário Macau SAR 999078 China; ^11^ Zhuhai Interventional Medical Center Zhuhai Precision Medical Center Zhuhai People's Hospital Zhuhai Hospital Affiliated with Jinan University Zhuhai Guangdong 519000 China; ^12^ Department of Vascular Surgery The Affiliated Hospital of Southwest Medical University Luzhou Sichuan 646000 China

**Keywords:** advanced breast cancer, drug screening, patient‐derived organoids, personalized therapy

## Abstract

Most breast cancers at an advanced stage exhibit an aggressive nature, and there is a lack of effective anticancer options. Herein, the development of patient‐derived organoids (PDOs) is described as a real‐time platform to explore the feasibility of tailored treatment for refractory breast cancers. PDOs are successfully generated from breast cancer tissues, including heavily treated specimens. The microtubule‐targeting drug‐sensitive response signatures of PDOs predict improved distant relapse‐free survival for invasive breast cancers treated with adjuvant chemotherapy. It is further demonstrated that PDO pharmaco‐phenotyping reflects the previous treatment responses of the corresponding patients. Finally, as clinical case studies, all patients who receive at least one drug predicate to be sensitive by PDOs achieve good responses. Altogether, the PDO model is developed as an effective platform for evaluating patient‐specific drug sensitivity in vitro, which can guide personal treatment decisions for breast cancer patients at terminal stage.

## Graphical abstract

Personalized therapies are urgently needed for patients with advanced breast cancers. Patient‐derived organoids (PDOs) can be generated from breast cancer tissues for the identification of anticancer drugs with high efficacy. PDO pharmaco‐phenotyping can not only reflect the previous treatment responses of patients, but also serve as an in‐time platform to guide tailored therapy for the refractory disease.

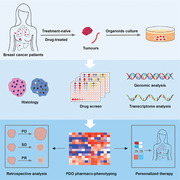

## 1 Introduction

Breast cancer is the most commonly diagnosed cancer among women worldwide.^[^ ^1^, ^2^, ^3^ ^]^ Despite the implementation of screening programs and improved therapeutics that have significantly enhanced the survival of breast cancer patients, ≈685 000 breast cancer‐related deaths were reported in 2020, making it the leading cause of cancer death in women.^[^ ^1^ ^]^ The choices of breast cancer therapeutic approaches are largely dependent on the clinical and molecular characteristics of the tumours.^[^ ^4^ ^]^ Systemic chemotherapy, endocrine therapy and human epidermal growth factor receptor 2 (HER2)‐targeted therapy are the most widely used means for breast cancer treatment.^[^ ^5^, ^6^ ^]^ Generally, these systemic medications are initially effective for most primary breast cancers. However, due to the heterogeneous features of breast cancer, a proportion of patients eventually develop recurrent disease and show resistance to the initial treatments.^[^ ^7^, ^8^ ^]^ Metastasis is another major challenge in the treatment of breast cancer. Metastatic breast cancers can be diagnosed at first presentation or at recurrence, and they frequently exhibit resistance to most of the standard treatment options.^[^ ^9^, ^10^ ^]^ The aggressive nature and high mortality rate of recurrent or metastatic breast cancer highlight the need for more precise treatment for these advanced diseases.

The high‐throughput capabilities of next‐generation sequencing (NGS) followed by bioinformatics analysis provide enormous insight into the individual genomic landscape of cancer and guide treatment decision‐making for optimal patient outcomes.^[^ ^11^, ^12^ ^]^ However, obstacles remain in translating the large amounts of oncogenomic data into information that is interpretable and accessible for clinical diagnosis and treatment.^[^ ^13^, ^14^ ^]^ Furthermore, even if potentially targetable genetic alterations are detected in the tumors, patients may still have no response to the corresponding drug treatment.^[^ ^15^ ^]^ Strategies to identify proper therapeutic options and increase treatment efficacy would be helpful for improving clinical outcomes. Patient‐derived xenograft (PDX) models have emerged as a powerful tool either to identify the best sensitive drugs for a specific patient or to understand the biological characteristics and evolutionary processes of the tumor.^[^ ^16^, ^17^ ^]^ Unfortunately, the low success rates (≈10–25% on average) and time consumption (6 months to 2 years) involved in establishing breast cancer PDX models in immunodeficient mice limit its application for preclinical and translational research.^[^ ^18^ ^]^

Recent studies have developed patient‐derived organoids (PDOs) as a promising model system for human cancers. It has been shown that PDOs, which can be derived from patient tumors in a short period of time with a high success rate, can accurately recapitulate the architecture and biological features of their parental tumors. To date, a broad spectrum of cancer PDO models have been successfully established, which can not only help to better understand cancer biology but also provide an ideal model for therapy efficacy testing in vitro.^[^ ^15^, ^19^, ^20^, ^21^, ^22^, ^23^, ^24^, ^25^, ^26^ ^]^ Indeed, several recent studies have demonstrated the good potential of this system to evaluate patient cancer responses to targeted therapy, chemotherapy, and radiotherapy.^[^ ^27^, ^28^, ^29^, ^30^ ^]^ Organoids derived from human breast cancer and breast normal tissues have been successfully established and characterized in previously studies. Their data indicated that organoid cultures are a valuable platform for studying breast cancer risk and drug development.^[^ ^21^, ^31^, ^32^, ^33^ ^]^ Nevertheless, whether breast cancer patients with advanced disease can benefit from PDOs based drug‐testing remain elusive. In the present study, we explore the application of breast cancer PDO as a promising platform for cancer precision medicine. Our case studies demonstrate that the PDO platform guided personal treatment is effective on patients with terminal breast cancer.

## 2 Results

### 2.1 Patient Samples and Clinical Data

A total of 132 breast cancer tissues were obtained from 125 female patients treated at five hospitals in China (data file S1, Figure S1, Supporting Information). Among these tumor tissues, 77 samples were acquired from treatment‐naïve patients, and 55 specimens were collected from patients who had been exposed to systemic anticancer therapy. The most common therapies that were administered directly to these patients after surgery were combination treatment with cyclophosphamide, epirubicin, and docetaxel. Twenty‐three samples were obtained from patients who exhibited resistance to the treatments they received. Metastatic breast cancer samples were obtained from tumor deposits in the lymph nodes (*n =* 6), lung (*n =* 6), chest wall (*n =* 5), bone (*n =* 3), liver (*n =* 4), and axillary fossa (*n =* 1). All patients underwent standard management, including endocrine therapy, local radiotherapy, curative surgical operations, and chemotherapy.

### 2.2 Establishment of Patient‐Derived Breast Cancer Organoids

**Figure 1:**
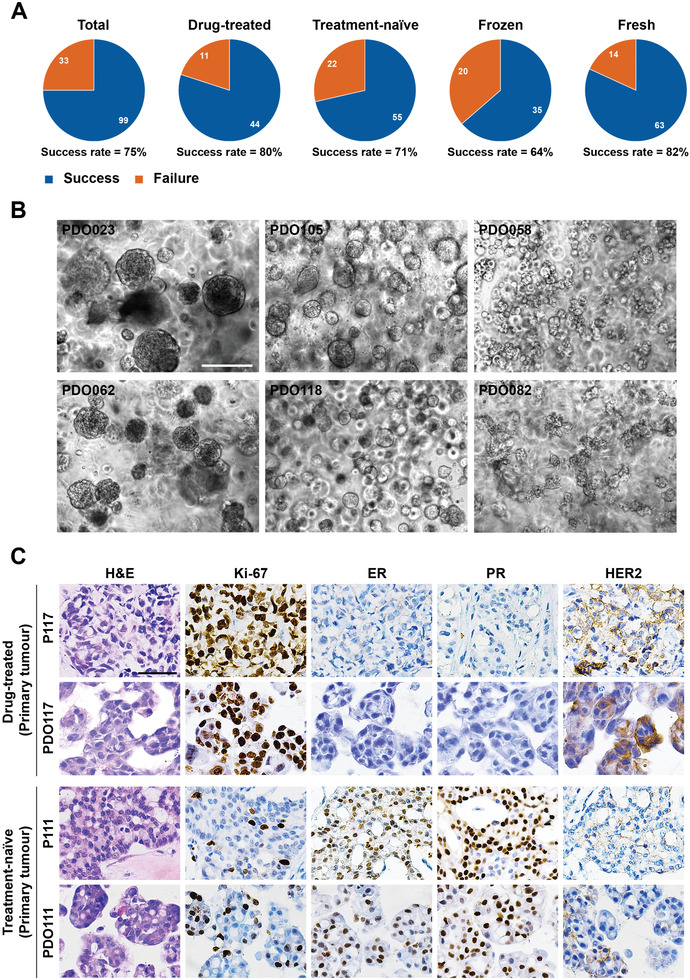
Establishing a biobank of breast cancer organoids for preclinical study. A) Isolation efficiency rate of PDOs from total, drug‐treated, treatment‐naïve, frozen, and fresh samples. B) Bright‐field microscopy images showing the representative phenotypes of breast cancer organoids. The left two images show cystic organoids (PDO023 and PDO062), the middle two images represent solid organoids (PDO105 and PDO118), the right top image has a grape‐like morphology (PDO058), and the right bottom image displays the morphology as a flower (PDO082). Scale bar: 200 µm. C) Histological and immunohistochemical images showing the organization structure and status of proliferation marker (Ki‐67) and breast cancer‐related markers (ER, PR, and HER2) in primary tumors and organoid lines. Scale bar: 50 µm. P117 and P111 represent tumor tissue, and PDO117 and PDO111 represent organoids.

To explore personalized therapy, we established the individual PDO model from human breast cancer tissues. In contrast to previous studies in which most PDOs were derived from treatment‐naïve primary tumors,^[^ ^21^ ^]^ we mainly focused on specimens from patients with high‐risk clinical features, including drug‐resistant and metastatic breast cancers. An important consideration is that improving therapeutic interventions for these patients with advanced disease is urgently needed. We eventually established 99 organoids from the 132 breast cancer samples with an overall success rate of 75% (**Figure** **1**A). The frozen samples had a lower success rate than the fresh samples, perhaps due to improper cryopreservation of these specimens at the beginning. As with technical progress, we have improved the success rate of the cryopreserved samples to a level similar to that of the fresh tissues. In particularly, most of the treatment‐naïve specimens used in this study were transported to the laboratory by cryopreservation (52 of 77 cases, 68%; data file S1, Supporting Information). During the clinical treatments, a proportion of the breast cancer patients underwent biopsies, and we also tried to generate organoids from these small specimens. The biopsied tumors were mainly collected from drug‐treated patients (14 of 17 cases, 82%), and 76% (13 of 17 cases) of them were from metastatic sites. Long‐term culturing of early generation is usually needed to establish organoid lines from these specimens. We finally generated 11 organoids from these biopsied tumors (*n =* 17) with a success rate of 65%. In addition, we found in some cases, the growth of PDOs was reduced after passage 2.

Morphological observation showed that most of the breast cancer organoids had a cystic or solid phenotype, and a few cases displayed a grape‐like morphology (Figure 1B). Of note, we generated an organoid line (PDO082) that presents flower‐like morphology from one biopsy of a lymph node specimen (P082) (Figure 1B). In addition, PDO082 also expanded well in 2D culture conditions, where they grew in a suspension status. We did not find significant morphological differences between the drug‐treated and treatment‐naïve organoid lines in this cohort. The organoid lines and the patient information are presented in data file S1, Supporting Information.

Overall, we successfully established a biobank of human breast cancer organoids derived from various types of breast cancers, including drug‐treated and treatment‐naïve, primary and metastatic, and resected and biopsied tumor tissues. Furthermore, we developed strategies for the collection and long‐term preservation of living human breast cancer tissues.

### 2.3 Breast Cancer PDOs Recapitulate the Histopathologic and Genetic Characteristics of Parental Tumors

Although previous studies have demonstrated that organoids derived from treatment‐naïve breast cancers recapitulate the histological features of parental tumors,^[^ ^21^ ^]^ whether the organoids derived from drug‐treated tumors also have this property remains to be determined. Histopathologic characteristics were compared in 12 pairs of breast cancer organoids (passage 1–2) and parental tumors. Haematoxylin and eosin (H&E) staining was performed to compare the morphological features of organoids and original tumors. We found that organoid lines established from drug‐treated tumors as well as lines from treatment‐naïve tumors inherited the histologic features of their parental tumor tissues, including the growth patterns and cellular and nuclear atypia (Figure 1C; Figure S2, Supporting Information). We subsequently evaluated the status of estrogen receptor (ER), progesterone receptor (PR), and human epidermal growth factor receptor 2 (HER2), the most common biomarkers for breast cancer,^[^ ^21^, ^34^ ^]^ and Ki‐67 on the organoids and matched tumors. Immunohistochemical staining results showed that the expression pattern of breast cancer markers was well preserved in both drug‐treated and treatment‐naïve tumor‐derived organoids (Figure 1C; Figure S2, Supporting Information). We also performed immunohistochemical analysis on four established long‐term culture breast cancer organoid lines. The status of ER/PR and HER2 were well conserved in PDO111, PDO141, and PDO118 after long‐term culture. However, although the status of PR and HER2 was conserved in PDO058, a proportion of cells changed from ER‐negative to ER‐positive. These results demonstrated that breast cancer organoids at early passage retained the histopathologic characteristics of the original tumors, regardless of whether they had received drug treatment. However, prolonging culture might yield some changes for PDOs.

**Figure 2:**
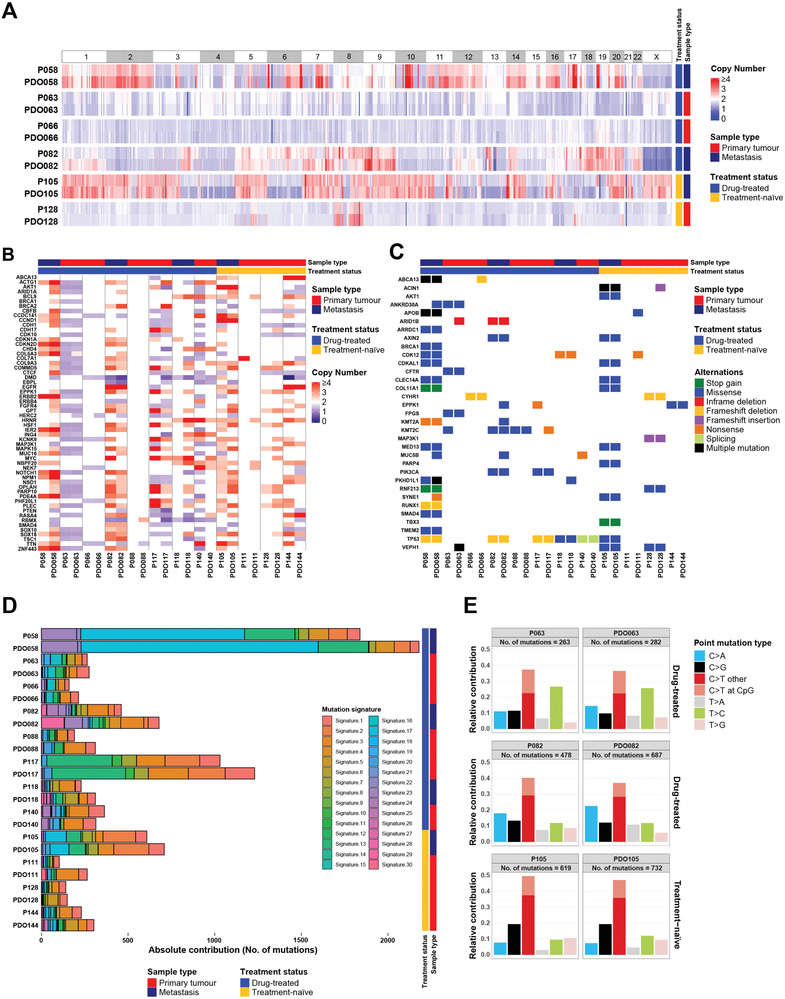
Breast cancer organoids recapitulate the genetic characterization of parental tumors. A) Comparison of the somatic copy number alteration landscape in 6 pairs of breast cancer organoids and parental tumors. Copy number application is shaded in red and copy number deletion is shaded in blue. B) Heatmap comparing the copy number of altered cancer genes in breast cancer organoids with that in parental tumors. C) Heatmap comparing the somatic mutated cancer genes in breast cancer organoids with those in parental tumors. D) Stacked bar chart displaying the proportional contribution of each COSMIC mutational signature in breast cancer organoids and parental tumors. E) Bar plots showing that the frequency of point mutation types is well conserved in the paired tumor tissue and organoid.

WES was performed in 12 pairs of breast cancer organoids (passage 1–2) and parental tumors to determine whether the breast cancer organoids derived from drug‐treated and treatment‐naïve tumors maintain the parental genomic features. Genome‐wide exome analysis of CNAs demonstrated that DNA copy number losses and gains of parental tumors were generally retained in the breast cancer organoid lines throughout the entire genome, including organoid lines that were generated from drug‐treated and metastatic tumors (**Figure** **2**A; Figure S3, Supporting Information). We observed that the number of CNA genes in organoids was generally more than that in the primary tumors (Table S1, Supporting Information). In three cases (P058, P063, and P128), more than 90% of the genes with copy number variations found in the primary tumors were preserved in organoids. However, in three cases (P088, P140, and P111), relatively small proportion of CNA genes were preserved in organoids. On average, 63.8% of the CNV genes found in the primary tumors were preserved in organoids. We further compared the CNA profiles of cancer‐related genes in organoids and corresponding breast cancers. Consistent with the genome‐wide results, breast cancer organoids recapitulated the CNA patterns of selected cancer‐related genes in an average of 66.8% of the original tumors (Figure 2B).

Next, we compared SNV genes in the parental tumors and organoids. On average, 82.1% of the selected cancer‐related genes found in the primary tumors were preserved in organoids (Figure 2C). Notably, mutations in breast cancer‐related genes, such as *TP53*, *PIK3CA*, *CTFR*, and *COL11A1*, were well retained in most breast cancer organoids. Of our samples, P058 is a chest wall metastatic breast cancer that has been heavily treated with multiple drugs. Importantly, most of the mutation loads and types of P058 were conserved in the corresponding organoids. We further analyzed base mutations in breast cancer tumors and organoids. The total mutational load and point mutation type of primary tumors were well conserved in the drug‐treated tumor‐derived organoids as well as the treatment‐naïve organoids (Figure 2D,E). The similarity of mutational signatures between organoids and the parental tumors are 87% (range from 67% to 99%) (Figure 2D). Similar to previous findings,^[^ ^21^ ^]^ we also observed that the relative contributions of point mutations were well conserved in the same organoid line after long‐term culture by comparing PDO058 at passage 2 and 15 (Figure S3C,D, Supporting Information).

In summary, organoids retain the genomic features of parental tumors to some extent, but there are also some differences between them. Higher number of CNA and SNV genes was generally found in organoids as compared with the parental tumors. One of the major reasons is that organoids might be derived from cancer initiating cells (CICs) or cancer stem cells (CSCs) that have amplification potential to grow up and differentiate to form PDOs. In addition, the primary tumor tissues should contain a certain proportion of multiple normal cells. Moreover, drug treatments could induce a certain proportion of dying cells in the tumors, these cells are difficult to continue to grow in the organoid culture. All these should contribute to the genomic heterogeneity between organoids and parental tumors.

### 2.4 Drug Screening in Breast Cancer PDOs

To assess the utility of breast cancer organoid lines as a real‐time platform for evaluating patient tumor drug responses, we performed drug sensitivity screening in vitro by using PDOs at early passage (passage 1–2). For the drug screening strategies, we first wanted to find the most sensitive drugs approved for breast cancer for each individual patient, and we also tried to develop potential candidates from non‐breast cancer drugs that could be applied for breast cancer treatment in the future. According to these considerations, we prepared a drug library containing 49 drugs, including 19 drugs for breast cancer and 35 drugs for the other types of cancer (data file S2, Supporting Information). Initial drug screening was performed on 20 organoid lines by using this drug panel with drug concentrations ranging from 20 × 10^−6^ m to 27.4 × 10^−9^ m (threefold dilution); drug sensitivity was represented by a concentration that inhibited 50% of cells (IC_50_). We then adjusted the ranges of some drugs based on their efficacy (data file S2, Supporting Information). The drug screen has high reproducibility in different experiments (Figure S4A,B, Supporting Information).

**Figure 3:**
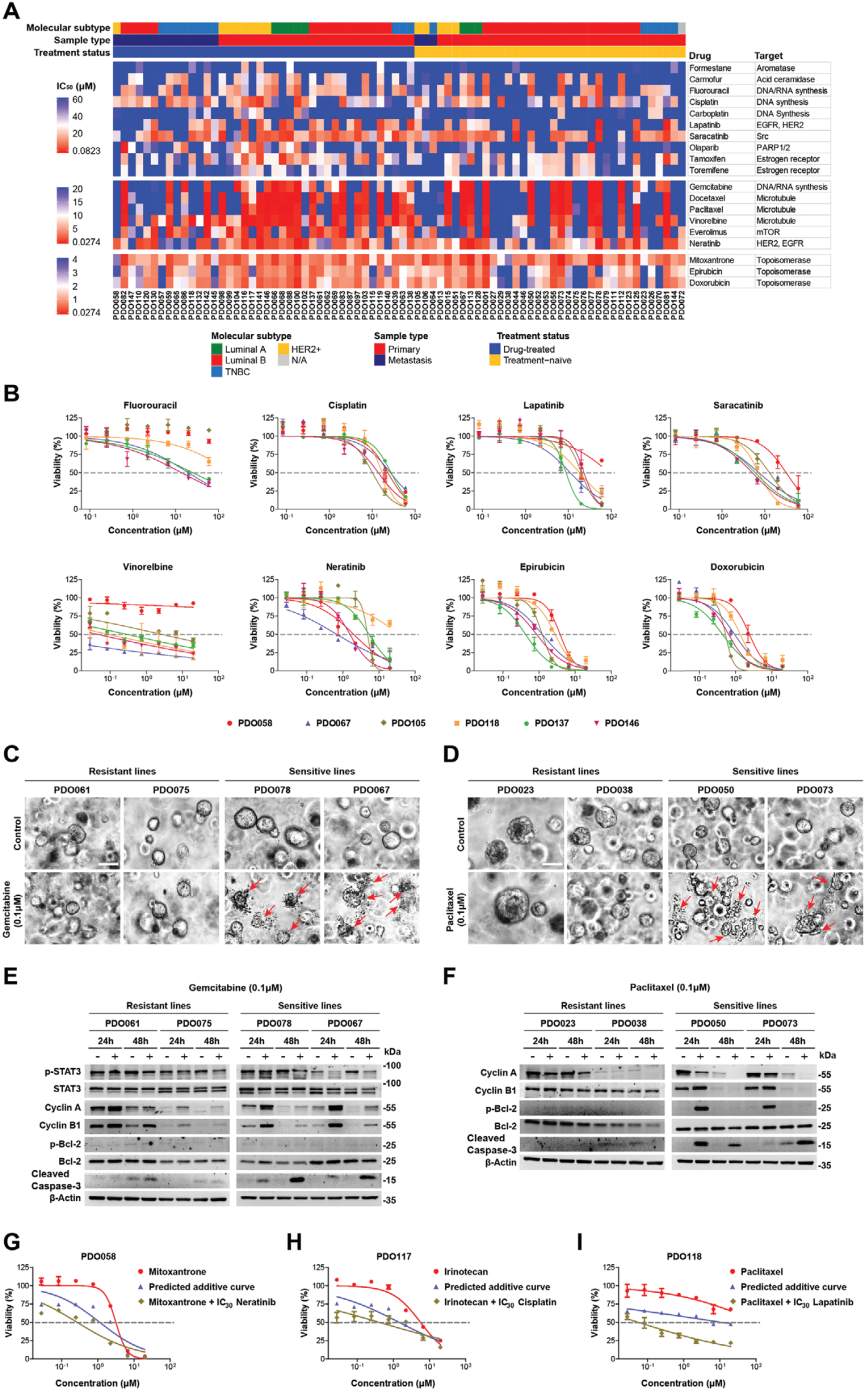
Breast cancer organoids serve as a platform for preclinical drug screening. A) Heatmap showing the IC_50_ values of 19 drugs for breast cancer in 76 organoid lines. They were divided into three groups according to the tested range of drug concentrations or IC_50_ values. Dose–response graphs of each group are indicated on the left. The tested drugs and their targets are listed on the right. The molecular subtype, sample type, and treatment status of the corresponding primary tumor are shown on the top graph. B) Representative drug response curves for PDO058, PDO067, PDO105, PDO118, PDO137, and PDO146. The results are expressed as the mean ± standard error of the mean (S.E.M.). C) Bright‐field microscopy images showing the morphological differences between gemcitabine‐resistant (PDO061 and PDO075) and ‐sensitive (PDO078 and PDO067) organoid lines after 48 h of treatment. The red arrows indicate structurally disrupted organoids. Scale bar: 50 µm. D) Bright‐field microscopy images showing the morphological differences between paclitaxel‐resistant (PDO023 and PDO038) and paclitaxel‐sensitive (PDO050 and PDO073) organoid lines after 48 h of treatment. The red arrows indicate structurally disrupted organoids. Scale bar: 50 µm. E) Western blot examinations were performed with the indicated antibodies of lysates from gemcitabine‐resistant (PDO061 and PDO075) and gemcitabine‐sensitive (PDO078 and PDO067) organoid lines after 24 and 48 h of treatment. F) Western blot examinations were performed with the indicated antibodies of lysates from paclitaxel‐resistant (PDO023 and PDO038) and ‐sensitive (PDO050 and PDO073) organoid lines after 24 and 48 h of treatment. G) Drug response curves of PDO058 treated with mitoxantrone alone, predicted additive curve, and mitoxantrone combined with IC_30_ neratinib. The results are expressed as the mean ± S.E.M. H) Drug response curves of PDO117 treated with irinotecan alone, predicted additive curve, and irinotecan combined with IC_30_ cisplatin. The results are expressed as the mean ± S.E.M. I) Drug response curves of PDO118 treated with paclitaxel alone, predicted additive curve, and paclitaxel combined with IC_30_ lapatinib. The results are expressed as the mean ± S.E.M.

Next, drug screening was performed on 76 breast cancer organoid lines (40 drug‐treated and 36 treatment‐naïve) by using the modified drug panel. The other two lines (PDO084 and PDO148) were tested with some drugs in the panel for insufficient amounts of cells. PDO pharmaco‐phenotyping showed striking variability in the PDO response to the drug treatment (**Figure** **3**A,B; Figure S4C, data file S2, Supporting Information). We found that breast cancer PDOs were consistently resistant to formestane and carboplatin but were commonly sensitive to treatment with mitoxantrone, epirubicin, and doxorubicin. In addition, we found that some non‐breast cancer drugs, including topotecan (topoisomerase I inhibitor) and bortezomib (proteasome inhibitor), had good responses in most organoid lines. To further evaluate the potential toxicity and feasibility of bortezomib for cancer treatment, we performed drug sensitivity testing of bortezomib on colon cancer and breast normal organoid lines. We found that the cancer organoids were more sensitive to the treatment of bortezomib than the normal organoid lines, which indicates the potential application of this drug for the treatment of different type of cancers (Figure S4D, Supporting Information). Notably, PDOs exhibited simultaneous sensitivity or resistance to nucleic acid synthesis inhibitors (gemcitabine, cladribine, and cytarabine) and microtubule inhibitors (docetaxel, paclitaxel, vinorelbine, ixabepilone, vincristine, and vinblastine). As compared with the primary tumors, organoids derived from metastatic tumors show a higher frequency of resistance to the microtubule‐ and EGFR‐targeting drugs. Of note, most of the organoids derived from metastatic tumors show resistance to palbociclib (CDK4/6 inhibitor) and cepharanthine (inhibiting TNF‐*α*‐mediated NF*κ*B stimulation). We did not find significantly different drug responses between the drug‐treated and treatment‐naïve groups. Multidrug resistance was found in a proportion of organoid lines, 13 of them were resistant to 75% of the tested drugs, while they were sensitive to no more than 3 drugs. In addition, multiple drug‐resistant tumor‐derived PDOs, such as PDO057, PDO058, and PDO115, showed high‐frequency resistance to a wide range of the tested drugs.

To confirm the distinct drug responses, we treated the resistant and sensitive organoid lines with DNA synthesis inhibitor (gemcitabine) or microtubule inhibitor (paclitaxel), respectively. After 24 h of treatment, we observed apoptotic vesicles surrounding the organoids in the sensitive lines, which was more obvious at 48 h (Figure 3C,D). However, resistant organoids maintained a smooth border during the treatment (Figure 3C,D). Western blotting analysis demonstrated that gemcitabine more efficiently reduced activated transcription factor (p‐STAT3), increased mitotic checkpoint protein (cyclin B1 and cyclin A), and increased pro‐apoptotic protein (cleaved caspase‐3) expression in the sensitive organoid lines (Figure 3E). With paclitaxel treatment, downregulation of cyclin A and upregulation of cyclin B1, p‐BCL, and cleaved caspase‐3 was observed in the sensitive organoid lines (Figure 3F). In Figure 3F, the growth rate of sensitive lines (PDO050 and PDO073) is significantly faster than that of the resistant lines (PDO023 and PDO038). The untreated organoids of the sensitive lines entered a state of growth arrest at 48 h due to the high cell density, which led to downregulation of the cyclins. However, in the paclitaxel treated group, this is mainly due to the death of sensitive organoids at 48 h.

To investigate the feasibility of using PDO platform to identify potential combination drug treatment for breast cancer patients, we further screened the anticancer drugs on three organoid lines by combining with the candidate drugs at the concentration of IC_30_. Based on this strategy, we identified the potential combination therapy for the tested organoid lines (Figure 3G–I), which may provide more choice of drug treatment for patients with advanced diseases.

### 2.5 The PDO Pharmaco‐Transcriptomic Signature in Response to Microtubule‐Targeting Drugs Reflects the Clinical Treatment Outcome of Breast Cancer Patients

**Figure 4:**
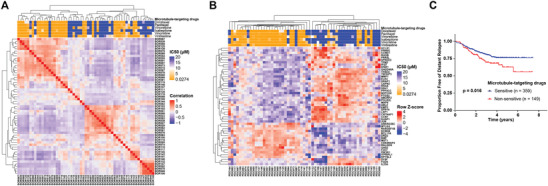
The microtubule‐targeting drug‐sensitive response signature derived from PDOs predicts breast cancer patients with an improved response to adjuvant chemotherapy. A) Correlation of PDO responses to microtubule‐targeting drugs and their gene expression. The top graph shows the IC_50_ values of 6 microtubule‐targeting drugs for 57 breast cancer PDOs. The bottom graph shows the hierarchical clustering of breast cancer organoids based on RNA‐seq expression data. Organoid lines are color‐coded by the correlation value. B) Breast cancer organoid lines are clustered by the microtubule‐targeting drug sensitive response signature. The IC_50_ values of the 6 microtubule‐targeting drugs for each PDO are shown on the top graph. C) Kaplan–Meier analysis of distant relapse‐free survival (DRFS) of patients (who received sequential taxane and anthracycline‐based regimens) with sensitive or non‐sensitive microtubule‐targeting drug response signatures. *p*‐values are from the log‐rank test.

Despite the prevalent historical and current applications, microtubule‐targeting drugs are only effective against a subgroup of breast cancers. Further insight into the mechanisms of action of microtubule‐targeting drugs is important to determine the application of these agents in breast cancers and improve overall clinical efficacy. We therefore performed RNA‐sequencing on 57 breast cancer organoid lines to assess their transcriptional profiles using six microtubule‐targeting drugs: docetaxel, paclitaxel, vinorelbine, ixabepilone, vincristine, and vinblastine. The correlation heatmap based on gene expression showed that organoid lines sensitive to microtubule‐targeting drugs tended to cluster together (**Figure** **4**A). Organoid lines that showed resistance or moderate responses to the microtubule‐targeting drugs clustered separately and were considerably more heterogeneous. These results suggest that the responses of breast cancer organoid lines to microtubule‐targeting drugs are highly correlated with their transcriptional profile.

We compared difference in gene expression between the microtubule‐targeting drug‐sensitive PDOs (sensitive to all six drugs) and ‐resistant PDOs (resistant to all six drugs), and the genes that significantly increased or decreased in the microtubule‐targeting drug‐sensitive group were defined as the sensitive response signature (Figure S5A,B, Supporting Information). Pathway enrichment analysis indicated the genes in this signature were highly associated with the regulation of response to drug, cell migration, apoptotic process, and focal adhesion. (Figure S5C, Supporting Information). By clustering the PDOs using the identified signatures, the PDOs could be grouped into sensitive and non‐sensitive classes (Figure 4B). To determine whether the PDO‐derived microtubule‐targeting drug‐sensitive response signature reflects the treatment responses of breast cancer patients, we obtained gene expression and associated follow‐up data from the Gene Expression Omnibus database [GEO: GSE25066].^[^ ^35^ ^]^ We obtained data from 508 patients, including 359 data for tumors with a drug‐sensitive response signature and 149 tumors with opposite features. We found that patients with enrichment for the microtubule‐targeting drug‐sensitive response signature had significantly higher distant relapse‐free survival than the resistant group (Figure 4C). The high correlation of PDO microtubule‐targeting drug‐sensitive response signatures with survival outcome suggested that PDO is a valuable platform for the development of novel therapeutic approaches and drugs for cancer patients.

### 2.6 PDO Pharmaco‐Phenotyping Reflects the Previous Treatment Responses of the Corresponding Patient

**Figure 5:**
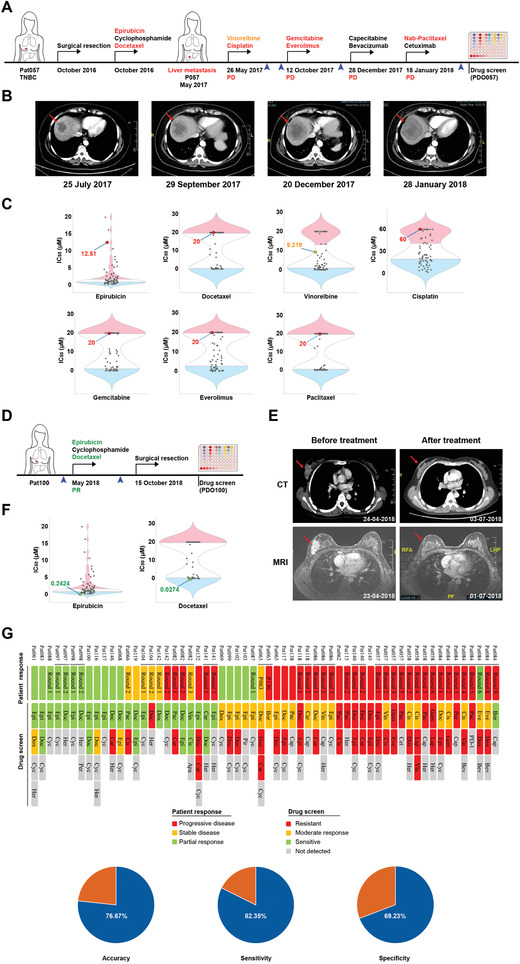
PDOs retain the previous treatment responses of breast cancer patients. A) Treatment procedure and responses of patient Pat057 from surgery till performing drug screening. The therapeutic agents in each round received by the patient are indicated above the arrow and color coded for in vitro drug screening results: intermediate and resistant drug responses are shaded in yellow and red, respectively. The blue arrow tips indicate the images taking time points in (B). PD, progressive disease. B) Tumor CT scan images show that the liver metastases of patient Pat057 continued to enlarge during the clinical treatment. The red arrows indicate tumors. C) Violin plot showing the distribution of IC_50_ values of the drugs in the 76 organoid lines, and IC_50_ values of the therapeutic agents received by patient Pat057 are indicated. The blue, white, and red parts represent the sensitive, intermediate, and resistant samples, respectively. D) Treatment procedure and responses of patient Pat100 before performing drug screening. The therapeutic agents received by the patient are indicated above the arrow and color coded for in vitro drug screening results: Sensitive drug responses are shaded in green. The blue arrow tips indicate the images taking time points in (E). PR, partial response. E) Tumor CT and MRI scan images show that the tumor size of patient Pat100 was significantly reduced after treatment. The red arrows indicate tumors. F) Violin plot showing the distribution of IC_50_ values of the drugs in the 76 organoid lines, and IC_50_ values of the therapeutic agents received by patient Pat100 are indicated. G) Drug screening results of breast cancer organoids matching the patients’ previous treatment outcomes. The treatment outcomes of Pat084 were compared with the drug screening results of PDO147 (the second organoid lines from Pat084). The top heatmap shows the patients’ clinical outcomes (35 patient cases) and organoid responses of the agents received by patients in each round of treatment. The bottom graph shows accuracy, sensitivity, and specificity of drug screening results matching the patients’ previous treatment outcomes. Drugs represented by abbreviations are summarized in data file S3, Supporting Information.

To determine whether PDO pharmaco‐phenotyping could represent the patients’ previous drug responses, we followed 35 patient cases with clear retrospective data. Patient Pat057 initially presented with grade 3 and triple‐negative breast cancer (TNBC) and sequentially underwent surgical resection and adjuvant chemotherapy (epirubicin, cyclophosphamide, and docetaxel). Three months after treatment, she developed recurrent disease with liver metastasis. She then sequentially received four rounds of additional treatment as follows: vinorelbine and cisplatin; gemcitabine and everolimus; capecitabine and bevacizumab; and nab‐paclitaxel (albumin‐bound paclitaxel) and cetuximab. The patient exhibited resistance to all these drug treatments and developed progressive disease (**Figure** **5**A,B). We derived organoids (PDO057) from the liver metastatic tissue of this patient at the end of these treatments and performed drug screening. The response to nab‐paclitaxel was assessed by using paclitaxel in vitro. Our pharmaco‐phenotyping results showed that PDO057 had a moderate response to vinorelbine and was resistant to all the other received drugs, which were included in our drug panel (Figure 5C).

Tumor sample P058 was collected from a patient (Pat058) who was diagnosed with HER2+ breast cancer. Patient P058 was treated with chemotherapy drugs (type unclear) and Herceptin before undergoing modified radical treatment, and the postoperative adjuvant agents were lip‐doxorubicin (liposomal doxorubicin), cisplatin, and Herceptin. One year after tumor resection, she developed widespread metastasis. T‐DM1 treatment resulted in stable disease, but the patient was resistant to combination treatment with vinorelbine, cisplatin, and lapatinib. Then, she was successively switched to combination treatments: 1) lipo‐paclitaxel (liposomal paclitaxel), capecitabine, and Herceptin and 2) gemcitabine, lapatinib, and Herceptin. With both treatments, the tumor initially showed a stable disease but eventually developed resistance. Last, she was involved in a phase I clinical trial of a HER2 monoclonal antibody (LZM005) and showed no response. After that, the chest wall metastatic tumor tissues were resected for organoid generation (Figure S6A,B, Supporting Information). Based on our pharmaco‐phenotyping results, PDO058 showed a moderate response to cisplatin but was resistant to doxorubicin, vinorelbine, lapatinib, paclitaxel, and gemcitabine (Figure S6C, Supporting Information).

In addition to these heavily treated patients, the patients who received fewer rounds of treatment were also included in our studies. P118 was a lung metastatic tumor that was resistant to the combined therapy: 1) epirubicin, cyclophosphamide, and docetaxel and 2) carboplatin and capecitabine (Figure S6D,E, Supporting Information). Our results demonstrated that PDO118 had moderate responses to carboplatin and resistance to epirubicin and docetaxel (Figure S6F, Supporting Information). Pat068, Pat083, and Pat100 received neoadjuvant chemotherapy (epirubicin, cyclophosphamide, and docetaxel), which resulted in partial response (Figure 5D,E; Figure S6G,H,J,K, Supporting Information). Pharmaco‐phenotyping results showed that PDO068, PDO083, and PDO100 were sensitive or moderately responsive to epirubicin, and all three lines were sensitive to docetaxel (Figure 5F; Figure S6I,L, Supporting Information).

The clinical outcomes of the 35 patients and their PDO pharmaco‐phenotyping results are summarized in Figure 5G. Notably, 71% of the treatments, which include at least one drug that was tested to be sensitive by PDOs, achieved stable disease or partial response. However, 93% of the treatments, which do not include any drug that was tested to be sensitive or moderately responsive by PDOs, developed progressive disease. Thus, the PDO pharmaco‐phenotyping results are highly correlated with clinical outcomes (*p* = 0.00009824), with 80.10% AUC (95% CI, 69.70–90.50%), 76.67% accuracy (95% CI, 65.00–86.67%), 82.35% sensitivity (95% CI, 41.12–94.12%), and 69.23% specificity (95% CI, 53.85–100.00%).

Some inconsistent cases can also be found in our studies. For example, patient Pat082 showed resistance to the combined treatment: 1) lipo‐paclitaxel and carboplatin and 2) docetaxel and epirubicin, while our pharmaco‐phenotyping results indicated that PDO082 was sensitive to paclitaxel, docetaxel, and epirubicin. Nonetheless, the patient achieved stable disease after third‐round treatment (vinorelbine, cisplatin, and apatinib), which was consistent with the sensitivity of PDO082 to vinorelbine and cisplatin. The inconsistency between the response to the first two rounds of treatments and the pharmaco‐phenotyping results might be due to the lack of tumor microenvironment of PDO or tumor evolution. Overall, our PDO chemosensitivity profile largely paralleled the retrospective clinical data of the corresponding patients.

### 2.7 PDO Pharmaco‐Phenotyping Predicates Patient‐Specific Sensitivities for Personalized Therapy

**Figure 6:**
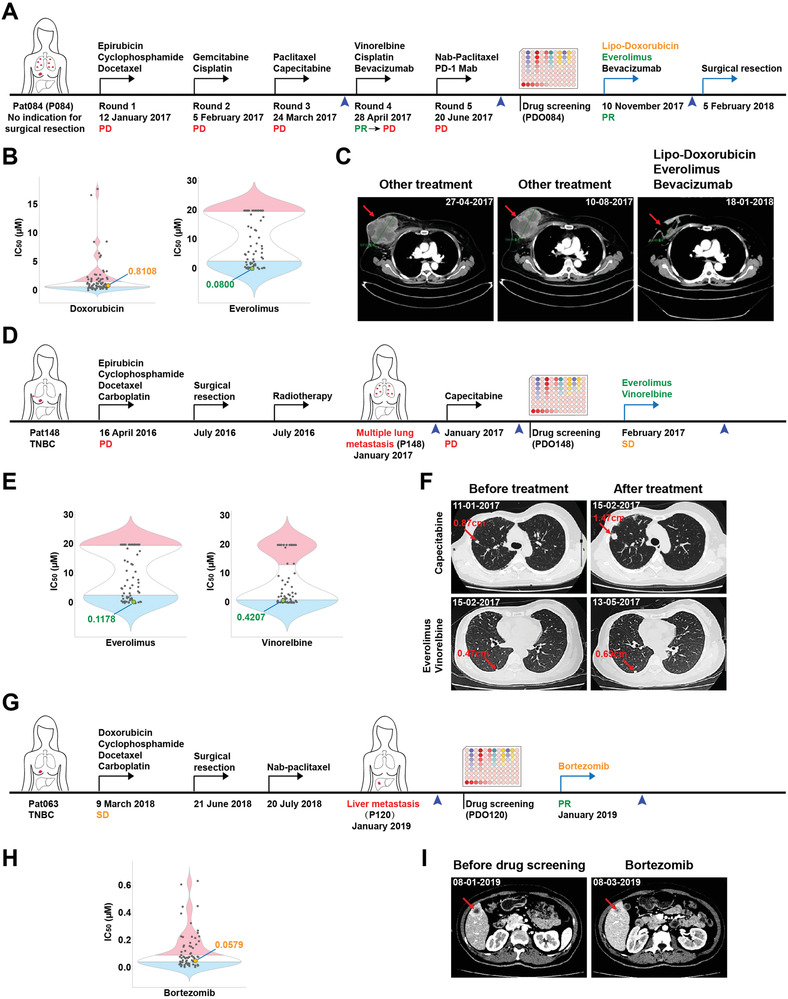
The PDO platform predicts personalized therapy for patients with advanced breast cancer. A) Treatment procedure and response of patient Pat084. The therapeutic agents in each round received by the patient are indicated above the arrow. The blue arrow tips indicate the images taking time points in (C). PD, progressive disease. PR, partial response. B) Violin plot showing the distribution of IC_50_ values of the drugs in the 76 organoid lines and IC_50_ values of the therapeutic agents received by patient Pat084 based on drug screening are indicated. The blue, white, and red parts represent the sensitive, intermediate, and resistant samples, respectively. C) Tumor CT scan images of patient Pat084 during the clinical treatment. The tumor showed resistance to all five rounds of therapy before drug screening, while it was especially sensitive to personalized therapy (lipo‐doxorubicin, everolimus, and bevacizumab). The red arrows indicate tumors. D) Treatment procedures and responses of patient Pat148. The therapeutic agents in each round received by the patient are indicated above the arrow. The blue arrow tips indicate the images taking time points in (F). PD, progressive disease. SD, stable disease. E) Violin plot showing the distribution of IC_50_ values of the drugs in the 76 organoid lines and IC_50_ values of the therapeutic agents received by patient Pat148 after drug screening are indicated. F) Tumor CT scan images of patient Pat148 before and after treatment with capecitabine or personalized therapy (everolimus and vinorelbine). The lung metastatic foci grew from 0.87 to 1.47 cm in 1 month when treated with capecitabine, while another lung metastatic foci enlarged from 0.47 to 0.69 cm during the 3 months of personalized therapy and resulted in stable disease. The red arrows indicate tumors. G) Treatment procedures and responses of patient Pat063. The therapeutic agents in each round received by the patient are indicated above the arrow. The blue arrow tips indicate the images taking time points in (I). SD, stable disease. PR, partial response. H) Violin plot showing the distribution of IC_50_ values of the drugs in the 76 organoid lines and IC_50_ values of the therapeutic agent received by patient Pat063 after drug screening is indicated. I) Tumor CT scan images of patient Pat063 before and after treatment with bortezomib. The red arrows indicate tumors.

One of the major objectives of this platform is the ability to predicate special sensitive drugs for each individual patient. Here, we mainly focused on multiple‐drug resistant and metastatic breast cancers. Patient Pat084 was diagnosed at a late stage with a large primary tumor (8.9 cm × 8.1 cm), multiple metastases of both lungs, and no indications for surgical resection. Moreover, the tumor (P084) was resistant to five rounds of treatment (**Figure** **6**A). Then, we generated 3D organoids (PDO084) and performed drug screening and found that doxorubicin, everolimus, and epirubicin were potential candidates for this patient. In addition, the patient participated in a commercial targeted‐sequencing service, and the results showed that there was a *PTEN* gene mutation in her tumor (the genomic sequence between base 599 of the coding region and base 5 of the intron 6 was replaced by GAAAT). This may lead to the loss function of *PTEN* gene, resulting in abnormal activation of the PI3K/AKT/mTOR pathway and suggesting that mTOR may be one of the effective therapeutic targets for this tumor.^[^ ^36^ ^]^ Based on these results and clinical practice guidelines, the patient was treated with lipo‐doxorubicin, everolimus, and bevacizumab. Of note, this combination treatment resulted in effective tumor regression with reduced tumor volume and necrosis of most lesions, which enabled a modified radical operation to be performed on the patient (Figure 6B,C).

Patient Pat148 developed TNBC before she came to the hospital. The patient experienced tumor progression on neoadjuvant chemotherapy (epirubicin, cyclophosphamide, docetaxel, and carboplatin). She was then treated with modified radical mastectomy and adjuvant radiation. Six months after treatment, she developed multiple lung metastases and showed resistance to capecitabine, and the length of one tumor foci grew from 0.87 to 1.47 cm in 1 month (Figure 6D,F). We generated organoids (PDO148) from one biopsied lung metastatic tumor and performed drug screening. Our results suggested that everolimus, mitoxantrone, and vinorelbine were potential candidates for this patient. According to clinical practice guidelines, the patient was switched to everolimus and vinorelbine for 3 months and achieved stable disease (Figure 6E,F).

Patient Pat063 carried TNBC and was treated with neoadjuvant chemotherapy (doxorubicin, cyclophosphamide, docetaxel, and carboplatin) before surgical resection. Thus, she was treated with one cycle of nab‐paclitaxel and was disease free for 7 months; then, she developed liver metastasis (Figure 6G). Organoids (PDO120) were established from the liver metastatic foci, and drug screening results indicated that temsirolimus, neratinib, and bortezomib were potential candidates for this patient. Based on our drug screening results, she was treated with bortezomib, a proteasome‐targeted drug that has been approved for mantle cell lymphoma and multiple myeloma but not for breast cancer. A significant reduction in tumor size was observed in the liver after the treatment (Figure 6H,I).

We totally apply this approach to guide the treatment of six patients, all of whom had advanced diseases with TNBC, drug‐resistant, or metastatic tumors (Figure 6, **Table** **1**; Figure S7, Supporting Information). Benefitted from these personalized therapies, three patients (Pat063, Pat084, and Pat138) achieved partial response, two patients (Pat086 and Pat148) achieved stable disease, and one patient (Pat081) is disease free till now. We followed up seven additional patient cases (Table 1; Figures S7,S8, Supporting Information). According to our data, 100% of the treatments (5/5), which include at least one drug that is predicated to be sensitive by PDOs, achieved partial response, stable disease, or long disease‐free survival. Of the patients who received at least one drug predicated to be moderately responsive by PDOs, 50% (3/6) of the patients developed progressive disease, one achieved partial response, and the other two achieved progression free survival of 11 and 17 months, respectively, after surgical resection of the tumors. For the remaining two treatments (Pat058 and Pat062) did not include any drug predicated to be sensitive or moderately responsive by PDOs, and both patients developed progressive disease. Overall, all 13 patients exhibited response predictable by our drug sensitive test. Our clinical case studies also indicate that the PDOs are most likely derived from CICs or CSCs, and the drug response obtained from PDOs should largely reflect that of primary tumors. Thus, our data suggest that PDOs could serve as a diagnostic platform to support and guide drug treatment against advanced breast cancer.

**Table 1:** Summary of PDO predicated drug‐responses and patients’ clinical outcomes

				PDO predicated responses of drugs received by patients			
Patient ID	Molecular subtype	Metastatic status	No. of resistant drugs before sampling	Sensitive	Moderate	Resistant	Clinical outcomes
Pat084 (P084)*	Luminal B	Multiple lung metastases	11	Everolimus	Doxorubicin	/	Partial response (Figure 6A–C)
Pat138*	TNBC	Lymph node	2	Gemcitabine	/	Carboplatin	Partial response (Figure S7D–F, Supporting Information)
Pat086*	TNBC	Axillary lymph node	7	Gemcitabine, Cisplatin	/	/	Stable disease (Figure S7A–C, Supporting Information)
Pat148*	TNBC	Lung	5	Vinorelbine, Everolimus	/	/	Stable disease (Figure 6D–F)
Pat081*	TNBC	/	Treatment‐naïve	Epirubicin, Docetaxel	/	/	Disease free survival > 28 months
Pat063 (P120)*	Luminal B	Liver	5	/	Bortezomib	/	Partial response (Figure 6G–I)
Pat059	TNBC	Lung	3	/	Docetaxel	/	Progression free survival = 11 months
Pat065	TNBC	Sternum and lymph node	3	/	Bortezomib	/	Progression free survival = 17 months
Pat057	TNBC	Multiple liver metastases	11	/	Vinorelbine	Gemcitabine, Paclitaxel	Progressive disease (Figure S7J–L, Supporting Information)
Pat105	HER2+	Multiple liver metastases	Treatment‐naïve	/	Epirubicin	Docetaxel	Progressive disease (Figure S7G–I, Supporting Information)
Pat132	TNBC	Chest wall	4	/	Vinorelbine	Carboplatin, Paclitaxel	Chest wall recurrence (Figure S7P–R, Supporting Information)
Pat058	HER2+	Widespread metastases	10	/	/	Irinotecan	Progressive disease (Figure S7M–O, Supporting Information)
Pat062	Luminal B	Axillary lymph node	2	/	/	Doxorubicin	Progressive disease

* Patients who received treatments guided by PDO pharmaco‐phenotyping results.

## 3 Discussion

Resistance to therapy is one of the major obstacles to the effective treatment of advanced breast cancer, which can occur either through preexisting or adaptive mechanisms.^[^ ^37^, ^38^ ^]^ Recent studies have revealed the potential mechanisms of drug resistance, including genomic mutations, activation of various signaling pathways, and alteration of the tumor microenvironment.^[^ ^39^, ^40^, ^41^, ^42^ ^]^ These data provide insights into the potential impact of drug treatment on the genomic alterations of breast cancer. However, little information can be applied for patient stratification or prediction of cancer treatment outcome. Models that faithfully recapitulate the characteristics of advanced breast cancer and their treatment responses are a critical unmet clinical need. Here, we attempted to exploit patient‐derived tumor organoids, a recently developed personalized model, as a real‐time platform to guide the clinical treatment of advanced breast cancers.

Employing the PDO culture system as an in vitro cancer model has been successfully established in various types of cancers, including human breast cancer.^[^ ^21^ ^]^ However, whether organoid technology can be successful with drug‐treated breast cancer, especially heavily treated tumor specimens, has not been explored. Generally, heavily treated patients show resistance to a wide range of drugs and thus have a more urgent need for personalized treatment. In this study, we demonstrated that breast cancer PDOs could be successfully established not only from the residual tissues of neoadjuvant chemotherapy‐treated patients but also from the tumor specimens of multiple‐drug‐resistant patients. In addition, we established a protocol for the collection and long‐term preservation of living human breast cancer tissues, which is important for samples that require long‐distance transport. Furthermore, regardless of whether the PDOs were generated from drug‐treated tumors or from treatment‐naïve tumors, their original histopathological and genomic characteristics were maintained to some extent.

Preclinical models are widely used for the discovery and preclinical testing of novel therapeutic strategies.^[^ ^43^ ^]^ Traditional 2D cell culture is one of the pioneering methodologies and has been widely used for high‐throughput drug testing. However, this model cannot mimic 3D growth in vivo and fails to adequately recapitulate the biology of the native tumor.^[^ ^43^, ^44^ ^]^ Advances in methods to generate PDO models offer the opportunity to establish a large biobank that more closely mirrors patient diseases, which can be adapted to identify subtype‐specific sensitive agents by using high‐throughput drug sensitivity testing. In this study, significant heterogeneity of drug responses was observed in the breast cancer PDO lines (Figure 3; Figure S4, Supporting Information), which is concordant with the diversity of clinical treatment responses. Our findings together with recently published data suggest that the cancer organoid lines generated from large cohorts of patients may become a valuable platform for the development of novel cancer drugs.^[^ ^45^, ^46^ ^]^ Furthermore, we developed organoid lines as a platform for drug repurposing strategies. Here, we screened 49 drugs, including 30 drugs that have not been approved for the treatment of breast cancer. Our results showed that most PDOs lines were sensitive to bortezomib (a proteasome inhibitor) and cantharidin (a protein/DNA/RNA synthesis inhibitor). Interestingly, in one of our recent studies, we found that broad drug resistance of cancers can be overcome by low‐dose bortezomib.^[^ ^47^ ^]^ EGFR‐targeted inhibitors (afatinib, gefitinib, erlotinib, and dacomitinib) and mTOR inhibitors (everolimus and temsirolimus) also had good responses in a portion of PDO lines. Our results suggest the potential application of these drugs for the treatment of breast cancer.

Transcriptional signatures derived from patients with different treatment outcomes have been shown to be robust prediction tools in many types of cancers.^[^ ^48^, ^49^, ^50^, ^51^ ^]^ However, these studies require a large cohort of patients to be recruited for long‐term follow‐up. In contrast, PDO pharmaco‐transcriptomic signatures can be successfully generated from the in vitro drug testing system in a short time frame. The signatures have also been shown to be useful for predicting chemotherapeutic drug responses in pancreatic ductal adenocarcinoma.^[^ ^24^ ^]^ In addition, the PDO pharmaco‐transcriptomic signatures may be conductive to the recruitment of patients with indications for drugs in clinical trial. Nevertheless, the correlation between these pharmaco‐transcriptomic signatures and clinical treatment outcomes requires further study and evaluation.

The major goal of this study was to investigate whether the PDO model could potentially be used as a companion diagnostic tool to predict the therapy response in a personalized manner for breast cancer patients, especially for patients with advanced disease. In this aspect, we first compared the prior treatment response of breast cancer patients with their PDO drug screening results. Our data revealed that most PDO lines established from residual tumors well recapitulated their clinically relevant responses to drug treatment. To go beyond the scope of previous studies, we applied the PDO platform to predict personalized therapy for individual patients. We investigated breast cancer cases with late‐stage disease in which standard treatment options had been exhausted. Efficient responses were observed in these patients who were treated with the effective agents identified by PDOs. Notably, patient Pat148, who was diagnosed with multiple‐drug resistant and multiple‐lung metastatic TNBC, remains alive more than 2 years after personalized therapy. A critical component of our functional pipeline for precision oncology is that PDOs can serve as real‐time patient‐derived models, that is, using PDOs at early passage (p1–p2), to test drug response for advanced breast cancer patients. With the improvement of experimental techniques, we can now establish organoid lines from surgically resected samples within 1 week and biopsy samples within 3 weeks, with an additional period of 6 days for finishing the drug screen. In this study, we completed drug testing on average 2.8 weeks. During this cycle, most patients have relatively stable disease and remain alive. So far, some previous studies about PDOs based drug testing should be the co‐clinical studies, in which PDOs were generated from patients enrolled in the clinical trial and treated with the agents to emulate clinical treatment responses. One of the prime limitations of co‐clinical study is that it is a correlative study rather than treatment guidance. In our study, in addition to co‐clinical study, we took a step further and applied PDOs to guide the treatment of breast cancer patients. Although our study, which involves a small cohort of patient treatment, suggests the potential feasibility of using PDOs as drug testing platform to guide personalized therapy for cancer patients, more patients should be recruited for further testing. Currently, we are planning to conduct prospective clinical trials to investigate whether the patients are clinical benefitted by this approach.

Although PDO models have the limitation of lacking an intact immune system, recent studies have developed a technological platform to evaluate the individual response to immunotherapy by co‐culture of peripheral blood lymphocytes with tumor organoids.^[^ ^52^, ^53^ ^]^ This is the approach we are currently exploring, together with some other issues yet to be addressed for the application of PDO models as precision medicine platforms. Some commonly used therapeutic agents, such as cyclophosphamide and capecitabine, need to be catalyzed by enzymes in the liver and converted to active products. Our ex vivo model cannot predict the in vivo treatment responses of these kinds of drugs. In addition, agents that target tumor angiogenesis or regulate hormone levels cannot be evaluated in this platform. Furthermore, the tumor microenvironment may also lead to the discrepancies between in vitro and in vivo drug responses.^[^ ^54^ ^]^

In summary, we have generated a breast cancer organoid biobank from tumor tissues with a broad spectrum of treatment statuses, disease stages, and molecular subtypes of breast cancers with a high success rate. We found that PDOs, even established from heavily treated breast cancers, were able to retain the histological and genetic features of their primary tumors to some extent. We further proved that the treatment response of primary tumors was also well retained in their PDO lines. More importantly, we developed the PDO model as a real‐time platform to guide clinical drug treatment for advanced breast cancer. Our study demonstrates that PDOs not only serve as a pre‐clinical model for broader cancer studies but also will provide personalized therapy recommendations for patients with advanced disease.

## 4 Experimental Section

### Human Specimens and Clinical Case Studies

Breast cancer and normal‐adjacent specimens and blood samples were obtained from five hospitals in China. This study was assessed and approved by the ethics committees of each respective institute: Affiliated Hospital of Southwest Medical University (Accreditation number: K2017042), First Affiliated Hospital of Sun Yat‐sen University (Accreditation number: ICE‐2017‐148), Sun Yat‐sen University Cancer Center (Accreditation number: 308‐2015‐001), Centro Hospitalar Conde de S. Januário (Accreditation number: BSERE16‐APP010‐FHS), and Zhuhai People's Hospital (Accreditation number: ZH2019‐03) (data file S1, Supporting Information). Informed consent was obtained prior to acquisition of samples from all donors. The clinical information of patients was obtained from the medical records system and summarized in data file S1, Supporting Information. All the surgically resected samples were separated into three parts for histology examination, genomic sequencing, and organoid generation. Tumor samples for organoid generation from the Affiliated Hospital of Southwest Medical University were minced into small pieces (1–3 mm^3^) in frozen medium (10% DMSO (Sigma Aldrich) + 90% FBS (GIBCO)) on ice, cryopreserved in liquid nitrogen by gradient cooling, and transported to the laboratory in dry ice. Tissue samples from the other hospitals were placed on ice in DMEM (GIBCO) supplemented with 10% FBS (GIBCO), 100 units mL^−1^ penicillin (GIBCO), and 100 µg mL^−1^ streptomycin (GIBCO) and transported to the laboratory within 6 h. Like the surgically resected samples, biopsy tissues with lengths over 30 mm (1 mm diameter) were separated into three parts, while smaller samples were used for organoid generation only.

This study was a co‐clinical study of a randomized phase III clinical trial (ClinicalTrials.gov identifier: NCT03006614): https://clinicaltrials.gov/ct2/show/NCT03006614. Samples obtained from this clinical trial were indicated in data file S1, Supporting Information.

Clinical trials guided by PDO Pharmaco‐phenotyping were intended to be clinical case studies. The trials were assessed and approved by the ethics committees of the hospitals. All patients provided written informed consent per the principles of the Declaration of Helsinki.

### Tissue Dissociation

For tissue dissociation, the fresh tissues were first minced into 1–3 mm^3^ pieces in a small volume of digestion buffer: DMEM/F12 medium (GIBCO) supplemented with 300 U mL^−1^ collagenase type III (Worthington), 100 U mL^−1^ hyaluronidase (Sigma Aldrich), 5% FBS (GIBCO), 5 µg mL^−1^ insulin (Sigma), 500 ng mL^−1^ hydrocortisone (Sigma Aldrich), 10 ng mL^−1^ EGF (PeproTech), and 20 ng mL^−1^ cholera toxin (Sigma Aldrich). Frozen tissues were thawed at 37 °C and spun down at 400 g for 4 min. According to the size of tissues, 6–12 mL digestion buffer was added to the tissue pieces. The tissues were digested on a shaker at 37 °C for 2–6 h with occasional pipetting until the visible pieces disappeared. Dissociated cell clusters were spun down at 400 g for 4 min, washed once with DMEM supplemented with 10% FBS, and spun down again at 400 g for 4 min. If the pellet showed a visible red color, erythrolysis was performed with RBC lysis buffer (eBiosciences) before the washing step.

### Organoid Culture and Passage

Dissociated cell clusters were resuspended in 50% cold Matrigel (Corning) and seeded in a prewarmed 6‐well plate (Corning) at 25 µL drops. The drops were solidified in a 37 °C and 5% CO_2_ incubator for 30 min, and then 2.5 mL organoid culture medium was added to each well and refreshed every 2–3 days. The previously established culture conditions were used with slight modifications.^[^ ^21^ ^]^ Briefly, the composition of the culture medium was Advanced DMEM/F12 medium supplemented with 30% Wnt3A conditional medium, 10% R‐spondin 1 conditional medium, 10% Noggin conditional medium, 5 × 10^−6^ m Y‐27632, 0.5 × 10^−6^ m SB202190, 0.5 × 10^−6^ m A83‐01, 5 ng mL^−1^ EGF, 5 × 10^−9^ m neuregulin‐1, 500 ng mL^−1^ hydrocortisone, 1.25 × 10^−3^ m *N*‐acetyl‐L‐cysteine, 15 × 10^−3^ m HEPES, 1× B27, 1× Glutamax, 5 × 10^−9^ m *β*‐estradiol, 1× insulin‐transferrin‐selenium‐sodium pyruvate, 0.5 µg mL^−1^ amphotericin B, 5 µg mL^−1^ gentamicin, and 5 µg mL^−1^ plasmocin (Table S2, Supporting Information). As compared with previously protocol, hydrocortisone, *β*‐estradiol, and insulin‐transferrin‐selenium‐sodium pyruvate were added to support the growth of cells. L Wnt‐3A cell line, R‐spondin 1 T‐REx‐293 cell line, and Noggin T‐REx‐293 cell line were used to prepare the conditional medium (Table S3, Supporting Information).

Passaging was performed every 1–3 weeks based on organoid density and size. After removing the culture medium, organoids were resuspended in cold 0.25% trypsin by pipetting, and then incubated at 37 °C for 4 min. Additional pipetting and incubation were performed if needed. Subsequently, the cells were spun down at 400 g for 4 min, washed with 6 mL DMEM medium, and spun down again. The cells were resuspended in Matrigel at a ratio of 1:3, seeded in the plates and cultured as described above. No significant difference was found in the longest time or passages between the cryopreserved or fresh tissues derived organoids. To generate frozen stocks, the trypsin‐digested organoids were suspended in frozen medium and cryopreserved in liquid nitrogen by gradient cooling. Organoid lines could be successfully recovered from the cryopreserved stocks and passaged in Matrigel. Most of the cancer tissues the authors received were very small or tiny, especially those from biopsy. In addition, the authors needed to finish drug screening in a short time frame, thus, a large proportion of organoids were used for drug testing. Only a very few cases, the authors had enough PDOs for doing different types of experiments. Thus, the authors had to set up a priority order using the PDOs, which was: 1) drug sensitive test; 2) RNA‐seq; 3) frozen; 4) DNA‐seq; 5) continuing passaging; 6) histological study; and 7) Western blot, etc. The organoids at the same passage were used for drug screening, whole exome sequencing, RNA sequencing, and histopathologic characteristics.

### Histology and Immunohistochemistry

Tissues were fixed in 4% paraformaldehyde and embedded in paraffin using standard protocols. For processing organoids, they were resuspended in cold PBS by pipetting, spun down, and fixed in 4% paraformaldehyde for 30 min. The fixed organoids were stained with haematoxylin before transfer to dehydration and paraffin embedding. Paraffin sections with a thickness of 5 µm were used for all analyses, H&E staining was carried out for histopathological evaluation. Immunohistochemistry was performed using the ELF 97 Immunohistochemistry Kit (ThermoFisher, E6600). Primary antibodies used in this study included Ki‐67 (CST, 9449S, 1:500), anti‐ER (Abcam, ab16660, 1:200), anti‐PR (Abcam, ab101688, 1:400), and anti‐HER2 (CST, 2165, 1:200). All images were captured on a Nikon microscope (Nikon, ECLIPSE, Ci).

### Whole Exome Sequencing

Genomic DNA samples were prepared with a DNeasy Blood & Tissue kit (QIAGEN) according to the manufacturer's instructions. A total amount of 1 µg genomic DNA per sample was input for library preparation after sonicating by a hydrodynamic shearing system (Covaris, Massachusetts, USA) to generate 180–280 bp fragments. The whole‐exome captured libraries were prepared using the Agilent SureSelect Human All Exon Kit (Agilent Technologies, CA, USA) following their protocols. The resulting products were qualified and quantified using the Agilent High Sensitivity DNA Kit on the Agilent 2100 Bioanalyzer. The libraries were clustered using TruSeq PE Cluster Kit v4‐cBot‐HS (Illumina, San Diego, USA) on a cBot Cluster Generation System and sequenced using Illumina NovaSeq 6000 with paired end 150 bp reads.

### Whole‐Exome Sequencing Data Analysis

Quality control of the DNA‐sequencing data was carried out using FastQC (v0.11.8). Adapter trimming was performed using Trim Galore (v0.5.0). Sequence reads were mapped against the human reference genome GRCh38 using Burrows‐Wheeler Alignment with maximal exact matches (BWA‐MEM) (v0.7.17).^[^ ^55^ ^]^ QC statistics were collected and summarized using Multiqc (v1.7).^[^ ^56^ ^]^ SAMtools (v0.1.9) was used to sort alignment files and collect statistics from bam files.^[^ ^57^, ^58^ ^]^ Data preprocessing was performed by the Genome Analysis ToolKit (v4.1.1.0) according to the best practice guidelines.^[^ ^59^, ^60^, ^61^ ^]^ Somatic mutations were detected by comparison of each cancer sample to the matched reference blood leukocytes or adjacent tissues. Somatic SNVs (single‐nucleotide variants) were detected using MuTect (v1.1.7).^[^ ^62^ ^]^ Somatic indels were detected using Strelka (v2.9.10) with supporting reads greater than 5.^[^ ^63^ ^]^ Annovar (v2018‐04‐16) was used to annotate the result with dbSNP (v150), dbNSFP (v3.3a), COSMIC database (v70), and 1000 Genomes (v201508).^[^ ^64^ ^]^ To detect somatic CNAs (copy number alterations), BAM files were analyzed for read‐depth variations using Control‐FREEC (v11.4) by comparing tumors or organoids to reference blood leukocytes or adjacent tissues, and Annovar (v2018‐04‐16) was used to annotate the result to acquire genes in specific regions.^[^ ^65^ ^]^ Mutational signature analysis was performed using the R package MutationalPatterns (v1.10.0) to calculate the optimal contribution of COSMIC signatures and determine the genomic context for all somatic SNVs in tumor tissues and organoid lines.^[^ ^66^ ^]^

### Drug Response Assay

Drug screening was performed by using the modified culture medium (Y‐27632, SB202190, and A83‐01 were removed from the complete culture medium), which does not affect the proliferation of PDOs during the screening period (6 days). This was mainly due to the consideration that these inhibitors might had some potential effects on special signaling pathways which may affect the results of drug screening. Organoids were dissociated into smaller clusters as described above and resuspended in 2.5% Matrigel/modified culture medium. Approximately 1500 cells in 36 µL were seeded in each well of the type‐I collagen gel pre‐coated 384‐well plate. Forty‐eight hours after seeding, 4 µL of a threefold dilution series (7‐point) of each compound was dispensed, and three technical replicates of each drug were tested on three plates. Bortezomib (1 × 10^−6^ m) and 0.2% DMSO were served as positive and negative control, respectively. After 4 days of drug incubation, cell viability was quantitated using the CellTiter‐Glo 2.0 assay (Promega) according to the manufacturer's instructions. The results were normalized to controls and expressed as percent cell viability. IC_50_ values were generated using GraphPad Prism 6. For some organoid lines resistant to specific drugs, their IC_50_ values exceeded the highest concentration of detection and were given the maximum concentration of the tested drug. In contrast, some organoid lines were very sensitive to specific drugs, and their IC_50_ values exceeded the lowest concentration of detection and were given the minimum concentration of the tested drug. In combination drug testing, the authors fixed the dose of one drug at IC_30_ (which was obtained from first round of drug testing) and another drug with gradient dilution. The predicted addictive curve was calculated by multiplying normalized mean cell viability of gradient diluted drug by mean cell viability of the fixed agent, which was 70%.

After obtaining IC_50_, the approach described by Tiriac et al. was used^[^ ^24^ ^]^ to determine if a PDO was sensitive or resistant to a drug. Drug sensitivity determined by this method was shown to have a high correlation with clinical treatment outcomes. Briefly, PDO lines to each drug were divided into three subgroups based on their IC_50_ values: sensitive group (lowest 33.3%), moderate response group (middle 33.3%), and resistant group (top 33.3%). Drugs that exhibited the maximum or lowest IC_50_ values in more than 33.3% of PDOs were classified as the sensitive or resistant group, respectively, and the remaining specimens were subdivided into two groups.

### Western Blotting

To obtain the drug‐treated organoids, dissociated cells were seeded in 6‐well plates at 15 µL drops. After 4 days of culture, the complete culture medium was replaced with fresh modified culture medium. Then, the organoids were treated with 0.1 × 10^−^ ^6^ m gemcitabine, 0.1 × 10^−6^ m paclitaxel, or 0.2% DMSO for 24 and 48 h. Organoids were resuspended in cold PBS by pipetting and spun down at 400 g and 4°C for 4 min, repeated twice for a total of 3 washing steps to remove the residual Matrigel. The harvested organoids were lysed in RIPA buffer (10 × 10^−3^ m Tris‐HCl pH 8, 140 × 10^−3^ m NaCl, 1 × 10^−3^ m EDTA, 0.5 × 10^−3^ m EGTA, 1% Triton, and 0.1% SDS) supplemented with phosphatase inhibitor cocktail (Sigma). The concentration of proteins was determined by the Enhanced BCA Protein Assay Kit (Pierce Biotechnology). In all, 30 µg of protein was resolved by SDS‐PAGE and transferred to PVDF membranes. Membranes were incubated with the following primary antibodies overnight at 4 °C: p‐Stat3 (CST, 9145S, 1:1000), Stat3 (CST, 9139S, 1:1000), cyclin A (Santa Cruz, sc‐751, 1:200), cyclin B1 (Santa Cruz, sc‐752, 1:200), p‐Bcl‐2 (CST, 2827S, 1:1000), Bcl‐2 (Proteintech, 12789‐1‐AP, 1:1000), cleaved caspase‐3 (CST, 9661S, 1:1000), and *β*‐actin (Sigma, A5316, 1:1000). Subsequently, the membranes were incubated with HRP‐conjugated goat anti‐rabbit (CST, 7074S, 1:5000) or anti‐mouse (CST, 7076S, 1:5000) IgG secondary antibodies at room temperature for 1 h. The antigen‐antibody reaction was detected using Immobilon Western Chemiluminescent HRP Substrate (Millipore) with a ChemiDoc Touch Imaging System (Bio‐Rad).

### RNA Sequencing

Total RNA from organoids was extracted using the RNeasy Micro Kit (Qiagen) following the manufacturer's instructions. The RNA samples were qualified and quantified by using the Agilent RNA 6000 Nano Kit on the Agilent 2100 Bioanalyzer, and high‐quality RNA with an RIN score > 7 was used for RNA library preparation. Five hundred nanograms of total RNA per sample were used as the starting material. Isolation, fragmentation, and priming of mRNA were performed by using the NEBNext Poly(A) mRNA Magnetic Isolation Module (NEB) according to the manufacturer's instructions. RNA‐seq libraries were prepared with the NEBNext Ultra II RNA Library Prep Kit for Illumina (NEB) following the manufacturer's protocols. The adaptor‐ligated libraries were enriched by eight cycles of polymerase chain reaction. Libraries were sequenced using the Illumina NovaSeq 6000 with paired end 150 bp reads.

### RNA Sequencing Analysis

FastQC (v0.11.8) was used on RNA sequencing data for quality control. Trim Galore (v0.5.0) was used to remove adapters. In addition, RNA reads were aligned to GRCh38 using STAR (v2.7.0e) and SAMtools (v0.1.9) was used to sort alignment files and collect statistics from bam files.^[^ ^57^, ^58^, ^67^ ^]^ Estimating the read count of each genomic feature was performed by featureCounts (v1.6.4).^[^ ^68^ ^]^ Differentially expressed genes (DEGs) between the microtubule‐targeting drug‐sensitive PDOs (sensitive to all six drugs) and ‐resistant PDOs (resistant to all six drugs) were identified by the edgeR package (v3.26.8).^[^ ^69^ ^]^ DEGs were filtered by FDR ≤ 0.01 and FC ≥ 2 and then mapped to cancer‐related signaling pathways.^[^ ^70^ ^]^ The Spearman correlation between PDO gene expression and the IC_50_ of the drugs were calculated, and DEGs with Spearman correlation higher than 0.3 were defined as the microtubule‐targeting drug‐sensitive response signature. To apply this signature to predict patients’ treatment responses, classifier training was performed using a linear support vector machine implemented in the R package “e1071” (v1.7‐3).^[^ ^71^ ^]^

### Clinical Outcome Evaluation

The clinical response of patients to drug treatments in this study was evaluated by computed tomography (CT) or magnetic resonance imaging (MRI) according to Response Evaluation Criteria in Solid Tumors (RECIST) version 1.1, which could be divided into complete response (CR), partial response (PR), stable disease (SD), and progressive disease (PD). CR and PR indicated that the treatments had a good response in patients.^[^ ^72^ ^]^ For patients with incurable locally advanced or metastatic breast cancer, the major purpose of clinical treatment was to improve survival and preserve quality of life, and SD outcomes are acceptable. Common Terminology Criteria for Adverse Event (CTCAE) version 4.0 was used to grade the toxicity of drug treatments.

### Quantification and Statistical Analysis

The dose‐response curves shown in Figure 3B,G–I, Figure S4A,B,D, Supporting Information were generated using Microsoft Excel and GraphPad Prism 6, and the results are expressed as the mean ± S.E.M. The *p*‐value reported in Figure 4C was obtained using the Kaplan–Meier log‐rank test. Sequence analysis details are described in the relevant methods sections.

The correlations of PDO pharmaco‐phenotyping results with the clinical outcomes (35 patient cases with clear retrospective data) were evaluated based on AUC, accuracy, sensitivity, and specificity (Figure 5G).^[^ ^30^ ^]^ Patients with PR or SD were classified as good response, while PD was classified as poor response. 1000 bootstrapping simulations were used to determine the distributions of out‐of‐bag correlation. The data were reported as mean and 95% confidence interval. Pearson's chi‐square test was used to assess the statistical significance. All the analyses were performed with R (4.0.4).

## Conflict of Interest

The authors declare no conflict of interest.

## Author Contributions

C.X.D. and P.C. conceived the study and designed experiments. C.X.D. directed the study. P.C. and H.S. processed tumor dissociation and primary cell isolation. P.C. and R.B.D. developed the culture protocol. P.C. generated and cultured organoids, performed drug response assays. P.C. and R.B.D. contributed to analyze drug response assay. P.C. prepared DNA and RNA libraries for sequencing. P.C. performed western blot experiment. P.C., H.P.L., and L.J.W. performed H&E staining and immunostaining. X.Z., X.Y.L., and J.M.Z. contributed to bioinformatic analysis protocol development. X.Z., X.Y.L., and P.C. contributed to bioinformatic analyses. L.L.Y., J.B., N.S., D.T.S., B.W., J.B.W., Z.H.Y., H.Y.W., B.Q.W., K.X., Y.L., S.Z.F., T.K.C., N.W.L., Y.Y.Q., Y.M., K.M., M.Z., J.L.B., and L.Z. coordinated clinical records and provided patient specimens. A.P.Z. and D.Y.T. contributed to statistical analysis. Y.L., Y.X.S., and L.L.Y. contributed clinical data. X.L.X., Y.L., and Y.X.S. contributed critical comments on the manuscript. C.X.D. and P.C. wrote the manuscript.

## Supporting information

Supporting Information

Supporting Information 1

Supporting Information 2

Supporting Information 3

## Data Availability

The data that support the findings of current study are available from the corresponding author upon reasonable request.

## References

[1] H. Sung , J. Ferlay , R. L. Siegel , M. Laversanne , I. Soerjomataram , A. Jemal , F. Bray , Ca‐Cancer J. Clin. 2021, 71, 209. 33538338 10.3322/caac.21660

[2] C. E. DeSantis , J. Ma , M. M. Gaudet , L. A. Newman , K. D. Miller , A. Goding Sauer , A. Jemal , R. L. Siegel , Ca‐Cancer J. Clin. 2019, 69, 438. 31577379 10.3322/caac.21583

[3] N. Harbeck , F. Penault‐Llorca , J. Cortes , M. Gnant , N. Houssami , P. Poortmans , K. Ruddy , J. Tsang , F. Cardoso , Nat. Rev. Dis. Primers 2019, 5, 66. 31548545 10.1038/s41572-019-0111-2

[4] S. Guiu , S. Michiels , F. Andre , J. Cortes , C. Denkert , A. Di Leo , B. T. Hennessy , T. Sorlie , C. Sotiriou , N. Turner , M. Van de Vijver , G. Viale , S. Loi , J. S. Reis‐Filho , Ann. Oncol. 2012, 23, 2997. 23166150 10.1093/annonc/mds586

[5] C. M. Perou , T. Sorlie , M. B. Eisen , M. van de Rijn , S. S. Jeffrey , C. A. Rees , J. R. Pollack , D. T. Ross , H. Johnsen , L. A. Akslen , O. Fluge , A. Pergamenschikov , C. Williams , S. X. Zhu , P. E. Lonning , A. L. Borresen‐Dale , P. O. Brown , D. Botstein , Nature 2000, 406, 747. 10963602 10.1038/35021093

[6] T. Sorlie , C. M. Perou , R. Tibshirani , T. Aas , S. Geisler , H. Johnsen , T. Hastie , M. B. Eisen , M. van de Rijn , S. S. Jeffrey , T. Thorsen , H. Quist , J. C. Matese , P. O. Brown , D. Botstein , P. E. Lonning , A. L. Borresen‐Dale , Proc. Natl. Acad. Sci. USA 2001, 98, 10869. 11553815 10.1073/pnas.191367098 PMC58566

[7] R. Marcotte , A. Sayad , K. R. Brown , F. Sanchez‐Garcia , J. Reimand , M. Haider , C. Virtanen , J. E. Bradner , G. D. Bader , G. B. Mills , D. Pe'er , J. Moffat , B. G. Neel , Cell 2016, 164, 293. 26771497 10.1016/j.cell.2015.11.062 PMC4724865

[8] P. Razavi , M. T. Chang , G. Xu , C. Bandlamudi , D. S. Ross , N. Vasan , Y. Cai , C. M. Bielski , M. T. A. Donoghue , P. Jonsson , A. Penson , R. Shen , F. Pareja , R. Kundra , S. Middha , M. L. Cheng , A. Zehir , C. Kandoth , R. Patel , K. Huberman , L. M. Smyth , K. Jhaveri , S. Modi , T. A. Traina , C. Dang , W. Zhang , B. Weigelt , B. T. Li , M. Ladanyi , D. M. Hyman , N. Schultz , M. E. Robson , C. Hudis , E. Brogi , A. Viale , L. Norton , M. N. Dickler , M. F. Berger , C. A. Iacobuzio‐Donahue , S. Chandarlapaty , M. Scaltriti , J. S. Reis‐Filho , D. B. Solit , B. S Taylor , J. Baselga , Cancer Cell 2018, 34, 427. 30205045 10.1016/j.ccell.2018.08.008 PMC6327853

[9] L. R. Yates , S. Knappskog , D. Wedge , J. H. R. Farmery , S. Gonzalez , I. Martincorena , L. B. Alexandrov , P. Van Loo , H. K. Haugland , P. K. Lilleng , G. Gundem , M. Gerstung , E. Pappaemmanuil , P. Gazinska , S. G. Bhosle , D. Jones , K. Raine , L. Mudie , C. Latimer , E. Sawyer , C. Desmedt , C. Sotiriou , M. R. Stratton , A. M. Sieuwerts , A. G. Lynch , J. W. Martens , A. L. Richardson , A. Tutt , P. E. Lonning , P. J. Campbell , Cancer Cell 2017, 32, 169. 28810143 10.1016/j.ccell.2017.07.005 PMC5559645

[10] E. Gobbini , M. Ezzalfani , V. Dieras , T. Bachelot , E. Brain , M. Debled , W. Jacot , M. A. Mouret‐Reynier , A. Goncalves , F. Dalenc , A. Patsouris , J. M. Ferrero , C. Levy , V. Lorgis , L. Vanlemmens , C. Lefeuvre‐Plesse , S. Mathoulin‐Pelissier , T. Petit , L. Uwer , C. Jouannaud , M. Leheurteur , M. Lacroix‐Triki , A. L. Cleaud , M. Robain , C. Courtinard , C. Cailliot , D. Perol , S. Delaloge , Eur. J. Cancer 2018, 96, 17. 29660596 10.1016/j.ejca.2018.03.015

[11] F. Meric‐Bernstam , A. Johnson , V. Holla , A. M. Bailey , L. Brusco , K. Chen , M. Routbort , K. P. Patel , J. Zeng , S. Kopetz , M. A. Davies , S. A. Piha‐Paul , D. S. Hong , A. K. Eterovic , A. M. Tsimberidou , R. Broaddus , E. V. Bernstam , K. R. Shaw , J. Mendelsohn , G. B. Mills , J. Natl. Cancer Inst. 2015, 107, djv098. 25863335 10.1093/jnci/djv098 PMC4651038

[12] H. Beltran , K. Eng , J. M. Mosquera , A. Sigaras , A. Romanel , H. Rennert , M. Kossai , C. Pauli , B. Faltas , J. Fontugne , K. Park , J. Banfelder , D. Prandi , N. Madhukar , T. Zhang , J. Padilla , N. Greco , T. J. McNary , E. Herrscher , D. Wilkes , T. Y. MacDonald , H. Xue , V. Vacic , A. K. Emde , D. Oschwald , A. Y. Tan , Z. Chen , C. Collins , M. E. Gleave , Y. Wang , D. Chakravarty , M. Schiffman , R. Kim , F. Campagne , B. D. Robinson , D. M. Nanus , S. T. Tagawa , J. Z. Xiang , A. Smogorzewska , F. Demichelis , D. S. Rickman , A. Sboner , O. Elemento , M. A. Rubin , JAMA Oncol. 2015, 1, 466. 26181256 10.1001/jamaoncol.2015.1313 PMC4505739

[13] W. C. Cheng , I. F. Chung , C. Y. Chen , H. J. Sun , J. J. Fen , W. C. Tang , T. Y. Chang , T. T. Wong , H. W. Wang , Nucleic Acids Res. 2014, 42, D1048. 24214964 10.1093/nar/gkt1025 PMC3965046

[14] M. Arnedos , C. Vicier , S. Loi , C. Lefebvre , S. Michiels , H. Bonnefoi , F. Andre , Nat. Rev. Clin. Oncol. 2015, 12, 693. 26196250 10.1038/nrclinonc.2015.123

[15] S. J. Hill , B. Decker , E. A. Roberts , N. S. Horowitz , M. G. Muto , M. J. Worley Jr. , C. M. Feltmate , M. R. Nucci , E. M. Swisher , H. Nguyen , C. Yang , R. Morizane , B. S. Kochupurakkal , K. T. Do , P. A. Konstantinopoulos , J. F. Liu , J. V. Bonventre , U. A. Matulonis , G. I. Shapiro , R. S. Berkowitz , C. P. Crum , A. D. D'Andrea , Cancer Discovery 2018, 8, 1404. 30213835 10.1158/2159-8290.CD-18-0474 PMC6365285

[16] M. Hidalgo , F. Amant , A. V. Biankin , E. Budinska , A. T. Byrne , C. Caldas , R. B. Clarke , S. de Jong , J. Jonkers , G. M. Maelandsmo , S. Roman‐Roman , J. Seoane , L. Trusolino , A. Villanueva , Cancer Discovery 2014, 4, 998. 25185190 10.1158/2159-8290.CD-14-0001 PMC4167608

[17] S. Aparicio , M. Hidalgo , A. L. Kung , Nat. Rev. Cancer 2015, 15, 311. 25907221 10.1038/nrc3944

[18] T. Murayama , N. Gotoh , Cells 2019, 8, 621. 10.3390/cells8060621 PMC6628218 31226846

[19] J. Roper , T. Tammela , N. M. Cetinbas , A. Akkad , A. Roghanian , S. Rickelt , M. Almeqdadi , K. Wu , M. A. Oberli , F. J. Sanchez‐Rivera , Y. K. Park , X. Liang , G. Eng , M. S. Taylor , R. Azimi , D. Kedrin , R. Neupane , S. Beyaz , E. T. Sicinska , Y. Suarez , J. Yoo , L. Chen , L. Zukerberg , P. Katajisto , V. Deshpande , A. J. Bass , P. N. Tsichlis , J. Lees , R. Langer , R. O. Hynes , J. Chen , A. Bhutkar , T. Jacks , O. H. Yilmaz , Nat. Biotechnol. 2017, 35, 569. 28459449 10.1038/nbt.3836 PMC5462879

[20] L. Broutier , G. Mastrogiovanni , M. M. Verstegen , H. E. Francies , L. M. Gavarro , C. R. Bradshaw , G. E. Allen , R. Arnes‐Benito , O. Sidorova , M. P. Gaspersz , N. Georgakopoulos , B. K. Koo , S. Dietmann , S. E. Davies , R. K. Praseedom , R. Lieshout , J. N. M. IJzermans , S. J. Wigmore , K. Saeb‐Parsy , M. J. Garnett , L. J. van der Laan , M. Huch , Nat. Med. 2017, 23, 1424. 29131160 10.1038/nm.4438 PMC5722201

[21] N. Sachs , J. de Ligt , O. Kopper , E. Gogola , G. Bounova , F. Weeber , A. V. Balgobind , K. Wind , A. Gracanin , H. Begthel , J. Korving , R. van Boxtel , A. A. Duarte , D. Lelieveld , A. van Hoeck , R. F. Ernst , F. Blokzijl , I. J. Nijman , M. Hoogstraat , M. van de Ven , D. A. Egan , V. Zinzalla , J. Moll , S. F. Boj , E. E. Voest , L. Wessels , P. J. van Diest , S. Rottenberg , R. G. J. Vries , E. Cuppen , H. Clevers , Cell 2018, 172, 373. 29224780 10.1016/j.cell.2017.11.010

[22] S. H. Lee , W. Hu , J. T. Matulay , M. V. Silva , T. B. Owczarek , K. Kim , C. W. Chua , L. J. Barlow , C. Kandoth , A. B. Williams , S. K. Bergren , E. J. Pietzak , C. B. Anderson , M. C. Benson , J. A. Coleman , B. S. Taylor , C. Abate‐Shen , J. M. McKiernan , H. Al‐Ahmadie , D. B. Solit , M. M. Shen , Cell 2018, 173, 515. 29625057 10.1016/j.cell.2018.03.017 PMC5890941

[23] X. Li , H. E. Francies , M. Secrier , J. Perner , A. Miremadi , N. Galeano‐Dalmau , W. J. Barendt , L. Letchford , G. M. Leyden , E. K. Goffin , A. Barthorpe , H. Lightfoot , E. Chen , J. Gilbert , A. Noorani , G. Devonshire , L. Bower , A. Grantham , S. MacRae , N. Grehan , D. C. Wedge , R. C. Fitzgerald , M. J. Garnett , Nat. Commun. 2018, 9, 2983. 30061675 10.1038/s41467-018-05190-9 PMC6065407

[24] H. Tiriac , P. Belleau , D. D. Engle , D. Plenker , A. Deschenes , T. D. D. Somerville , F. E. M. Froeling , R. A. Burkhart , R. E. Denroche , G. H. Jang , K. Miyabayashi , C. M. Young , H. Patel , M. Ma , J. F. LaComb , R. L. D. Palmaira , A. A. Javed , J. C. Huynh , M. Johnson , K. Arora , N. Robine , M. Shah , R. Sanghvi , A. B. Goetz , C. Y. Lowder , L. Martello , E. Driehuis , N. LeComte , G. Askan , C. A. Iacobuzio‐Donahue , H. Clevers , L. D. Wood , R. H. Hruban , E. Thompson , A. J. Aguirre , B. M. Wolpin , A. Sasson , J. Kim , M. Wu , J. C. Bucobo , P. Allen , D. V. Sejpal , W. Nealon , J. D. Sullivan , J. M. Winter , P. A. Gimotty , J. L. Grem , D. J. DiMaio , J. M. Buscaglia , P. M. Grandgenett , J. R. Brody , M. A. Hollingsworth , G. M. O'Kane , F. Notta , E. Kim , J. M. Crawford , C. Devoe , A. Ocean , C. L. Wolfgang , K. H. Yu , E. Li , C. R. Vakoc , B. Hubert , S. E. Fischer , J. M. Wilson , R. Moffitt , J. Knox , A. Krasnitz , S. Gallinger , D. A. Tuveson , Cancer Discovery 2018, 8, 1112. 29853643 10.1158/2159-8290.CD-18-0349 PMC6125219

[25] H. H. N. Yan , H. C. Siu , S. Law , S. L. Ho , S. S. K. Yue , W. Y. Tsui , D. Chan , A. S. Chan , S. Ma , K. O. Lam , S. Bartfeld , A. H. Y. Man , B. C. H. Lee , A. S. Y. Chan , J. W. H. Wong , P. S. W. Cheng , A. K. W. Chan , J. Zhang , J. Shi , X. Fan , D. L. W. Kwong , T. W. Mak , S. T. Yuen , H. Clevers , S. Y. Leung , Cell Stem Cell 2018, 23, 882. 30344100 10.1016/j.stem.2018.09.016

[26] M. Kim , H. Mun , C. O. Sung , E. J. Cho , H. J. Jeon , S. M. Chun , D. J. Jung , T. H. Shin , G. S. Jeong , D. K. Kim , E. K. Choi , S. Y. Jeong , A. M. Taylor , S. Jain , M. Meyerson , S. J. Jang , Nat. Commun. 2019, 10, 3991. 31488816 10.1038/s41467-019-11867-6 PMC6728380

[27] G. Vlachogiannis , S. Hedayat , A. Vatsiou , Y. Jamin , J. Fernandez‐Mateos , K. Khan , A. Lampis , K. Eason , I. Huntingford , R. Burke , M. Rata , D. M. Koh , N. Tunariu , D. Collins , S. Hulkki‐Wilson , C. Ragulan , I. Spiteri , S. Y. Moorcraft , I. Chau , S. Rao , D. Watkins , N. Fotiadis , M. Bali , M. Darvish‐Damavandi , H. Lote , Z. Eltahir , E. C. Smyth , R. Begum , P. A. Clarke , J. C. Hahne , M. Dowsett , J. de Bono , P. Workman , A. Sadanandam , M. Fassan , O. J. Sansom , S. Eccles , N. Starling , C. Braconi , A. Sottoriva , S. P. Robinson , D. Cunningham , N. Valeri , Science 2018, 359, 920. 29472484 10.1126/science.aao2774 PMC6112415

[28] K. Ganesh , C. Wu , K. P. O'Rourke , B. C. Szeglin , Y. Zheng , C. G. Sauve , M. Adileh , I. Wasserman , M. R. Marco , A. S. Kim , M. Shady , F. Sanchez‐Vega , W. R. Karthaus , H. H. Won , S. H. Choi , R. Pelossof , A. Barlas , P. Ntiamoah , E. Pappou , A. Elghouayel , J. S. Strong , C. T. Chen , J. W. Harris , M. R. Weiser , G. M. Nash , J. G. Guillem , I. H. Wei , R. N. Kolesnick , H. Veeraraghavan , E. J. Ortiz , I. Petkovska , A. Cercek , K. O. Manova‐Todorova , L. B. Saltz , J. A. Lavery , R. P. DeMatteo , J. Massagué , P. B. Paty , R. Yaeger , X. Chen , S. Patil , H. Clevers , M. F. Berger , S. W. Lowe , J. Shia , P. B. Romesser , L. E. Dow , J. Garcia‐Aguilar , C. L. Sawyers , J. J. Smith , Nat. Med. 2019, 25, 1607. 31591597 10.1038/s41591-019-0584-2 PMC7385919

[29] S. N. Ooft , F. Weeber , K. K. Dijkstra , C. M. McLean , S. Kaing , E. van Werkhoven , L. Schipper , L. Hoes , D. J. Vis , J. van de Haar , W. Prevoo , P. Snaebjornsson , D. van der Velden , M. Klein , M. Chalabi , H. Boot , M. van Leerdam , H. J. Bloemendal , L. V. Beerepoot , L. Wessels , E. Cuppen , H. Clevers , E. E. Voest , Sci. Transl. Med. 2019, 11, eaay2574. 31597751 10.1126/scitranslmed.aay2574

[30] Y. Yao , X. Xu , L. Yang , J. Zhu , J. Wan , L. Shen , F. Xia , G. Fu , Y. Deng , M. Pan , Q. Guo , X. Gao , Y. Li , X. Rao , Y. Zhou , L. Liang , Y. Wang , J. Zhang , H. Zhang , G. Li , L. Zhang , J. Peng , S. Cai , C. Hu , J. Gao , H. Clevers , Z. Zhang , G. Hua , Cell Stem Cell 2020, 26, 17. 31761724 10.1016/j.stem.2019.10.010

[31] N. Goldhammer , J. Kim , V. Timmermans‐Wielenga , O. W. Petersen , Breast Cancer Res. 2019, 21, 141. 31829259 10.1186/s13058-019-1233-x PMC6907265

[32] J. M. Rosenbluth , R. C. J. Schackmann , G. K. Gray , L. M. Selfors , C. M. Li , M. Boedicker , H. J. Kuiken , A. Richardson , J. Brock , J. Garber , D. Dillon , N. Sachs , H. Clevers , J. S. Brugge , Nat. Commun. 2020, 11, 1711. 32249764 10.1038/s41467-020-15548-7 PMC7136203

[33] E. Campaner , A. Zannini , M. Santorsola , D. Bonazza , C. Bottin , V. Cancila , C. Tripodo , M. Bortul , F. Zanconati , S. Schoeftner , G. Del Sal , Cancers 2020, 12, 3869. 10.3390/cancers12123869 PMC7770601 33371412

[34] X. Dai , L. Xiang , T. Li , Z. Bai , J. Cancer 2016, 7, 1281. 27390604 10.7150/jca.13141 PMC4934037

[35] C. Hatzis , L. Pusztai , V. Valero , D. J. Booser , L. Esserman , A. Lluch , T. Vidaurre , F. Holmes , E. Souchon , H. Wang , M. Martin , J. Cotrina , H. Gomez , R. Hubbard , J. I. Chacon , J. Ferrer‐Lozano , R. Dyer , M. Buxton , Y. Gong , Y. Wu , N. Ibrahim , E. Andreopoulou , N. T. Ueno , K. Hunt , W. Yang , A. Nazario , A. DeMichele , J. O'Shaughnessy , G. N. Hortobagyi , W. F. Symmans , JAMA, J. Am. Med. Assoc. 2011, 305, 1873. 10.1001/jama.2011.593 PMC5638042 21558518

[36] F. Janku , D. S. Hong , S. Fu , S. A. Piha‐Paul , A. Naing , G. S. Falchook , A. M. Tsimberidou , V. M. Stepanek , S. L. Moulder , J. J. Lee , R. Luthra , R. G. Zinner , R. R. Broaddus , J. J. Wheler , R. Kurzrock , Cell Rep. 2014, 6, 377. 24440717 10.1016/j.celrep.2013.12.035 PMC4409143

[37] J. A. Gallaher , P. M. Enriquez‐Navas , K. A. Luddy , R. A. Gatenby , A. R. A. Anderson , Cancer Res. 2018, 78, 2127. 29382708 10.1158/0008-5472.CAN-17-2649 PMC5899666

[38] J. M. Balko , J. M. Giltnane , K. Wang , L. J. Schwarz , C. D. Young , R. S. Cook , P. Owens , M. E. Sanders , M. G. Kuba , V. Sanchez , R. Kurupi , P. D. Moore , J. A. Pinto , F. D. Doimi , H. Gomez , D. Horiuchi , A. Goga , B. D. Lehmann , J. A. Bauer , J. A. Pietenpol , J. S. Ross , G. A. Palmer , R. Yelensky , M. Cronin , V. A. Miller , P. J. Stephens , C. L. Arteaga , Cancer Discovery 2014, 4, 232. 24356096 10.1158/2159-8290.CD-13-0286 PMC3946308

[39] C. Kim , R. Gao , E. Sei , R. Brandt , J. Hartman , T. Hatschek , N. Crosetto , T. Foukakis , N. E. Navin , Cell 2018, 173, 879. 29681456 10.1016/j.cell.2018.03.041 PMC6132060

[40] M. Nedeljkovic , A. Damjanovic , Cells 2019, 8, 957. 10.3390/cells8090957 PMC6770896 31443516

[41] C. Denkert , G. von Minckwitz , S. Darb‐Esfahani , B. Lederer , B. I. Heppner , K. E. Weber , J. Budczies , J. Huober , F. Klauschen , J. Furlanetto , W. D. Schmitt , J. U. Blohmer , T. Karn , B. M. Pfitzner , S. Kummel , K. Engels , A. Schneeweiss , A. Hartmann , A. Noske , P. A. Fasching , C. Jackisch , M. van Mackelenbergh , P. Sinn , C. Schem , C. Hanusch , M. Untch , S. Loibl , Lancet Oncol. 2018, 19, 40. 29233559 10.1016/S1470-2045(17)30904-X

[42] G. V. Echeverria , Z. Ge , S. Seth , X. Zhang , S. Jeter‐Jones , X. Zhou , S. Cai , Y. Tu , A. McCoy , M. Peoples , Y. Sun , H. Qiu , Q. Chang , C. Bristow , A. Carugo , J. Shao , X. Ma , A. Harris , P. Mundi , R. Lau , V. Ramamoorthy , Y. Wu , M. J. Alvarez , A. Califano , S. L. Moulder , W. F. Symmans , J. R. Marszalek , T. P. Heffernan , J. T. Chang , H. Piwnica‐Worms , Sci. Transl. Med. 2019, 11, eaav0936. 30996079 10.1126/scitranslmed.aav0936 PMC6541393

[43] B. A. Chabner , J. Natl. Cancer Inst. 2016, 108, djv388. 26755050 10.1093/jnci/djv388

[44] A. Li , J. Walling , Y. Kotliarov , A. Center , M. E. Steed , S. J. Ahn , M. Rosenblum , T. Mikkelsen , J. C. Zenklusen , H. A. Fine , Mol. Cancer Res. 2008, 6, 21. 18184972 10.1158/1541-7786.MCR-07-0280

[45] C. Pauli , B. D. Hopkins , D. Prandi , R. Shaw , T. Fedrizzi , A. Sboner , V. Sailer , M. Augello , L. Puca , R. Rosati , T. J. McNary , Y. Churakova , C. Cheung , J. Triscott , D. Pisapia , R. Rao , J. M. Mosquera , B. Robinson , B. M. Faltas , B. E. Emerling , V. K. Gadi , B. Bernard , O. Elemento , H. Beltran , F. Demichelis , C. J. Kemp , C. Grandori , L. C. Cantley , M. A. Rubin , Cancer Discovery 2017, 7, 462. 28331002 10.1158/2159-8290.CD-16-1154 PMC5413423

[46] L. Li , H. Knutsdottir , K. Hui , M. J. Weiss , J. He , B. Philosophe , A. M. Cameron , C. L. Wolfgang , T. M. Pawlik , G. Ghiaur , A. J. Ewald , E. Mezey , J. S. Bader , F. M. Selaru , JCI Insight 2019, 4, e121490. 10.1172/jci.insight.121490 PMC6413833 30674722

[47] F. Shao , X. Lyu , K. Miao , L. Xie , H. Wang , H. Xiao , J. Li , Q. Chen , R. Ding , P. Chen , F. Xing , X. Zhang , G. H. Luo , W. Zhu , G. Cheng , N. W. Lon , S. E. Martin , G. Wang , G. Chen , Y. Dai , C. X. Deng , Adv. Sci. 2020, 7, 2001914. 10.1002/advs.202001914 PMC7709997 33304752

[48] F. Cardoso , L. J. van't Veer , J. Bogaerts , L. Slaets , G. Viale , S. Delaloge , J. Y. Pierga , E. Brain , S. Causeret , M. DeLorenzi , A. M. Glas , V. Golfinopoulos , T. Goulioti , S. Knox , E. Matos , B. Meulemans , P. A. Neijenhuis , U. Nitz , R. Passalacqua , P. Ravdin , I. T. Rubio , M. Saghatchian , T. J. Smilde , C. Sotiriou , L. Stork , C. Straehle , G. Thomas , A. M. Thompson , J. M. van der Hoeven , P. Vuylsteke , R. Bernards , K. Tryfonidis , E. Rutgers , M. Piccart , MINDACT Investigators , N. Engl. J. Med. 2016, 375, 717. 27557300 10.1056/NEJMoa1602253

[49] J. A. Sparano , R. J. Gray , D. F. Makower , K. I. Pritchard , K. S. Albain , D. F. Hayes , C. E. Geyer Jr. , E. C. Dees , M. P. Goetz , J. A. Olson Jr. , T. Lively , S. S. Badve , T. J. Saphner , L. I. Wagner , T. J. Whelan , M. J. Ellis , S. Paik , W. C. Wood , P. M. Ravdin , M. M. Keane , H. L. Gomez Moreno , P. S. Reddy , T. F. Goggins , I. A. Mayer , A. M. Brufsky , D. L. Toppmeyer , V. G. Kaklamani , J. L. Berenberg , J. Abrams , G. W. Sledge Jr. , N. Engl. J. Med. 2018, 379, 111. 29860917 10.1056/NEJMoa1804710 PMC6172658

[50] S. G. Zhao , S. L. Chang , D. E. Spratt , N. Erho , M. Yu , H. A. Ashab , M. Alshalalfa , C. Speers , S. A. Tomlins , E. Davicioni , A. P. Dicker , P. R. Carroll , M. R. Cooperberg , S. J. Freedland , R. J. Karnes , A. E. Ross , E. M. Schaeffer , R. B. Den , P. L. Nguyen , F. Y. Feng , Lancet Oncol. 2016, 17, 1612. 27743920 10.1016/S1470-2045(16)30491-0

[51] R. Nicolle , O. Gayet , P. Duconseil , C. Vanbrugghe , J. Roques , M. Bigonnet , Y. Blum , N. Elarouci , L. Armenoult , M. Ayadi , A. de Reynies , F. Puleo , J. Augustin , J. F. Emile , M. Svrcek , T. Arsenijevic , P. Hammel , M. Giovannini , P. Grandval , L. Dahan , V. Moutardier , M. Gilabert , J. L. Van Laethem , J. B. Bachet , J. Cros , J. Iovanna , N. J. Dusetti , Ann. Oncol. 2021, 32, 250. 33188873 10.1016/j.annonc.2020.10.601

[52] K. K. Dijkstra , C. M. Cattaneo , F. Weeber , M. Chalabi , J. van de Haar , L. F. Fanchi , M. Slagter , D. L. van der Velden , S. Kaing , S. Kelderman , N. van Rooij , M. E. van Leerdam , A. Depla , E. F. Smit , K. J. Hartemink , R. de Groot , M. C. Wolkers , N. Sachs , P. Snaebjornsson , K. Monkhorst , J. Haanen , H. Clevers , T. N. Schumacher , E. E. Voest , Cell 2018, 174, 1586. 30100188 10.1016/j.cell.2018.07.009 PMC6558289

[53] C. M. Cattaneo , K. K. Dijkstra , L. F. Fanchi , S. Kelderman , S. Kaing , N. van Rooij , S. van den Brink , T. N. Schumacher , E. E. Voest , Nat. Protoc. 2020, 15, 15. 31853056 10.1038/s41596-019-0232-9 PMC7610702

[54] A. A. Duarte , E. Gogola , N. Sachs , M. Barazas , S. Annunziato , J. R. de Ruiter , A. Velds , S. Blatter , J. M. Houthuijzen , M. van de Ven , H. Clevers , P. Borst , J. Jonkers , S. Rottenberg , Nat. Methods 2018, 15, 134. 29256493 10.1038/nmeth.4535

[55] H. Li , R. Durbin , Bioinformatics 2009, 25, 1754. 19451168 10.1093/bioinformatics/btp324 PMC2705234

[56] P. Ewels , M. Magnusson , S. Lundin , M. Kaller , Bioinformatics 2016, 32, 3047. 27312411 10.1093/bioinformatics/btw354 PMC5039924

[57] H. Li , B. Handsaker , A. Wysoker , T. Fennell , J. Ruan , N. Homer , G. Marth , G. Abecasis , R. Durbin , 1000 Genome Project Data Processing Subgroup , Bioinformatics 2009, 25, 2078. 19505943 10.1093/bioinformatics/btp352 PMC2723002

[58] H. Li , Bioinformatics 2011, 27, 2987. 21903627 10.1093/bioinformatics/btr509 PMC3198575

[59] G. A. Van der Auwera , M. O. Carneiro , C. Hartl , R. Poplin , G. Del Angel , A. Levy‐Moonshine , T. Jordan , K. Shakir , D. Roazen , J. Thibault , E. Banks , K. V. Garimella , D. Altshuler , S. Gabriel , M. A. DePristo , Curr. Protoc. Bioinf. 2013, 43, 11 10 1. 10.1002/0471250953.bi1110s43 PMC4243306 25431634

[60] M. A. DePristo , E. Banks , R. Poplin , K. V. Garimella , J. R. Maguire , C. Hartl , A. A. Philippakis , G. del Angel , M. A. Rivas , M. Hanna , A. McKenna , T. J. Fennell , A. M. Kernytsky , A. Y. Sivachenko , K. Cibulskis , S. B. Gabriel , D. Altshuler , M. J. Daly , Nat. Genet. 2011, 43, 491. 21478889 10.1038/ng.806 PMC3083463

[61] A. McKenna , M. Hanna , E. Banks , A. Sivachenko , K. Cibulskis , A. Kernytsky , K. Garimella , D. Altshuler , S. Gabriel , M. Daly , M. A. DePristo , Genome Res. 2010, 20, 1297. 20644199 10.1101/gr.107524.110 PMC2928508

[62] K. Cibulskis , M. S. Lawrence , S. L. Carter , A. Sivachenko , D. Jaffe , C. Sougnez , S. Gabriel , M. Meyerson , E. S. Lander , G. Getz , Nat. Biotechnol. 2013, 31, 213. 23396013 10.1038/nbt.2514 PMC3833702

[63] C. T. Saunders , W. S. Wong , S. Swamy , J. Becq , L. J. Murray , R. K. Cheetham , Bioinformatics 2012, 28, 1811. 22581179 10.1093/bioinformatics/bts271

[64] K. Wang , M. Li , H. Hakonarson , Nucleic Acids Res. 2010, 38, e164. 20601685 10.1093/nar/gkq603 PMC2938201

[65] V. Boeva , T. Popova , K. Bleakley , P. Chiche , J. Cappo , G. Schleiermacher , I. Janoueix‐Lerosey , O. Delattre , E. Barillot , Bioinformatics 2012, 28, 423. 22155870 10.1093/bioinformatics/btr670 PMC3268243

[66] F. Blokzijl , R. Janssen , R. van Boxtel , E. Cuppen , Genome Med. 2018, 10, 33. 29695279 10.1186/s13073-018-0539-0 PMC5922316

[67] A. Dobin , C. A. Davis , F. Schlesinger , J. Drenkow , C. Zaleski , S. Jha , P. Batut , M. Chaisson , T. R. Gingeras , Bioinformatics 2013, 29, 15. 23104886 10.1093/bioinformatics/bts635 PMC3530905

[68] Y. Liao , G. K. Smyth , W. Shi , Bioinformatics 2014, 30, 923. 24227677 10.1093/bioinformatics/btt656

[69] M. D. Robinson , D. J. McCarthy , G. K. Smyth , Bioinformatics 2010, 26, 139. 19910308 10.1093/bioinformatics/btp616 PMC2796818

[70] F. Sanchez‐Vega , M. Mina , J. Armenia , W. K. Chatila , A. Luna , K. C. La , S. Dimitriadoy , D. L. Liu , H. S. Kantheti , S. Saghafinia , D. Chakravarty , F. Daian , Q. Gao , M. H. Bailey , W. W. Liang , S. M. Foltz , I. Shmulevich , L. Ding , Z. Heins , A. Ochoa , B. Gross , J. Gao , H. Zhang , R. Kundra , C. Kandoth , I. Bahceci , L. Dervishi , U. Dogrusoz , W. Zhou , H. Shen , P. W. Laird , G. P. Way , C. S. Greene , H. Liang , Y. Xiao , C. Wang , A. Iavarone , A. H. Berger , T. G. Bivona , A. J. Lazar , G. D. Hammer , T. Giordano , L. N. Kwong , G. McArthur , C. Huang , A. D. Tward , M. J. Frederick , F. McCormick , M. Meyerson , E. M. Van Allen , A. D. Cherniack , G. Ciriello , C. Sander , N. Schultz , Cell 2018, 173, 321.29625050

[71] M. David , D. Evgenia , H. Kurt , W. Andreas , L. Friedrich , *e1071: Misc Functions of the Department of Statistics, Probability Theory Group (Formerly: E1071)*, TU Wien, Vienna, Austria, R package version 1.7‐3, https://CRAN.R‐project.org/package=e1071. 2019.

[72] E. A. Eisenhauer , P. Therasse , J. Bogaerts , L. H. Schwartz , D. Sargent , R. Ford , J. Dancey , S. Arbuck , S. Gwyther , M. Mooney , L. Rubinstein , L. Shankar , L. Dodd , R. Kaplan , D. Lacombe , J. Verweij , Eur. J. Cancer 2009, 45, 228. 19097774 10.1016/j.ejca.2008.10.026

