# Supplementary material for: Patient‐Derived Organoids Can Guide Personalized‐Therapies for Patients with Advanced Breast Cancer
Source: Adv Sci (Weinh). 2021 Oct 4;8(22):2101176. doi: 10.1002/advs.202101176 (PMC8596108; doi:10.1002/advs.202101176)
Supplement: Supplementary file 1 — Supporting Information [file ADVS-8-2101176-s004.pdf]

© 2021 Wiley-VCH GmbH

ADVANCED SCIENCE Open Access

Supporting Information

for *Adv. Sci.*, DOI: 10.1002/advs.202101176

Patient-Derived Organoids Can Guide Personalized-Therapies for Patients with Advanced Breast Cancer

Ping Chen, Xu Zhang, Renbo Ding, Linglin Yang, Xueying Lyu, Jianming Zeng, Josh Haipeng Lei, Lijian Wang, Jiong Bi, Nan Shao, Ditian Shu, Bin Wu, Jingbo Wu, Zhihui Yang, Haiyan Wang, Biqiong Wang, Kang Xiong, Yun Lu, Shaozhi Fu, Tak Kan Choi, Ng Wai Lon, Aiping Zhang, Dongyang Tang, Yingyao Quan, Ya Meng, Kai Miao, Heng Sun, Ming Zhao, Jiaolin Bao, Lei Zhang, Xiaoling Xu, Yanxia Shi, Ying Lin,* and Chuxia Deng**

Supplementary Materials for
Patient-derived organoids can guide personalized-therapies for patients with advanced breast cancer

Ping Chen, Xu Zhang, Renbo Ding, Linglin Yang, Xueying Lyu, Jianming Zeng, Josh Haipeng Lei, Lijian Wang, Jiong Bi, Nan Shao, Ditian Shu, Bin Wu, Jingbo Wu, Zhihui Yang, Haiyan Wang, Biqiong Wang, Kang Xiong, Yun Lu, Shaozhi Fu, Choi Tak Kan, Ng Wai Lon, Aiping Zhang, Dongyang Tang, Yingyao Quan, Ya Meng, Kai Miao, Heng Sun, Ming Zhao, Jiaolin Bao, Lei Zhang, Xiaoling Xu, Yanxia Shi[#], Ying Lin[#], Chuxia Deng[#]

#Correspondence Author. Email: shiyx@sysucc.org.cn (Y.X.S.); linying3@mail.sysu.edu.cn (Y.L.); cxdeng@umac.mo (C.X.D.)

The PDF file includes:

Figure S1. Subtypes and represent histology of breast cancer.
Figure S2. Histological and immunohistochemical analysis of breast cancer organoids and parental tumours.
Figure S3. Comparison of genomic landscape between breast cancer organoids and parental tumours.
Figure S4. Drug response analyses of breast cancer organoids.
Figure S5. The microtubule-targeting drug-sensitive response signature in breast cancer organoids.
Figure S6. PDOs retain previous treatment responses of corresponding breast cancer patients.
Figure S7. The PDOs platform predicts drug-responses and patients' clinical outcomes.
Figure S8. Summary of PDO predicated drug-responses and patients' clinical outcomes in Table 1.

Table S1. The number of CNA genes in tumours and organoids.
Table S2. The composition of the organoid culture medium.
Table S3. Cell lines used in this study.
Table S4. Antibodies used in immunohistochemistry and western blotting assays.
Table S5. Drugs tested in this study.
Table S6. Software used in this study.

Other Supplementary Material for this manuscript includes the following:

Data file S1 (Microsoft Excel format). Clinical characteristics and treatment outcomes of the breast cancer cohort.

Data file S2 (Microsoft Excel format). Abbreviations of therapeutic agents and approach.

Data file S3 (Microsoft Excel format). IC_{50} values of each drug in the breast cancer organoid lines.

Figure S1.

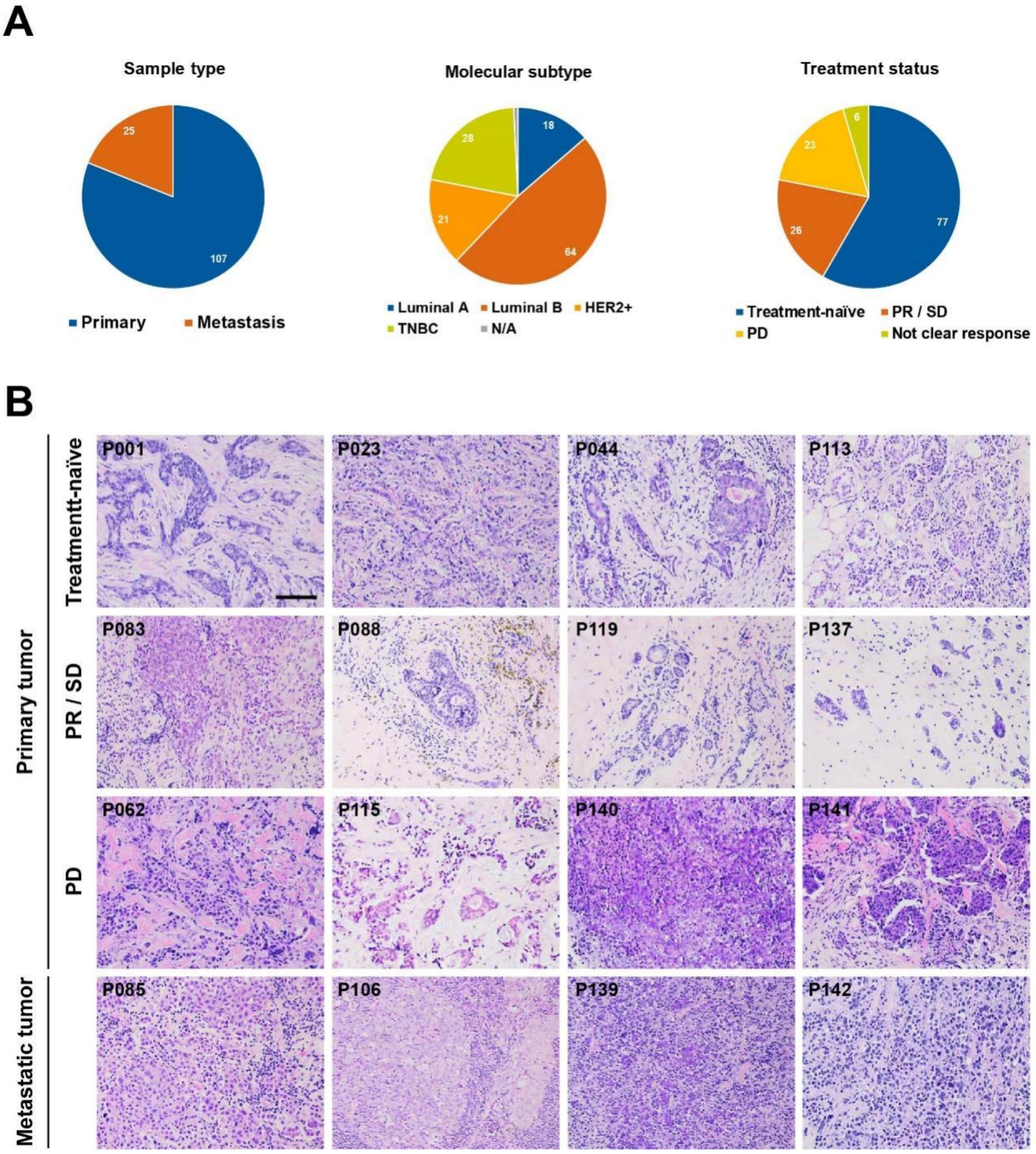

Figure S1. Subtypes and represent histology of breast cancer.

(A) Summary of breast cancer samples in this study according to their molecular subtype, metastatic and treatment status. PR, partial response. SD, stable disease. PD, progressive disease.

(B) Representative histological sections of breast cancers from different groups. PR, partial response. SD, stable disease. PD, progressive disease. Scale bar, 100 μm.

Figure S2.

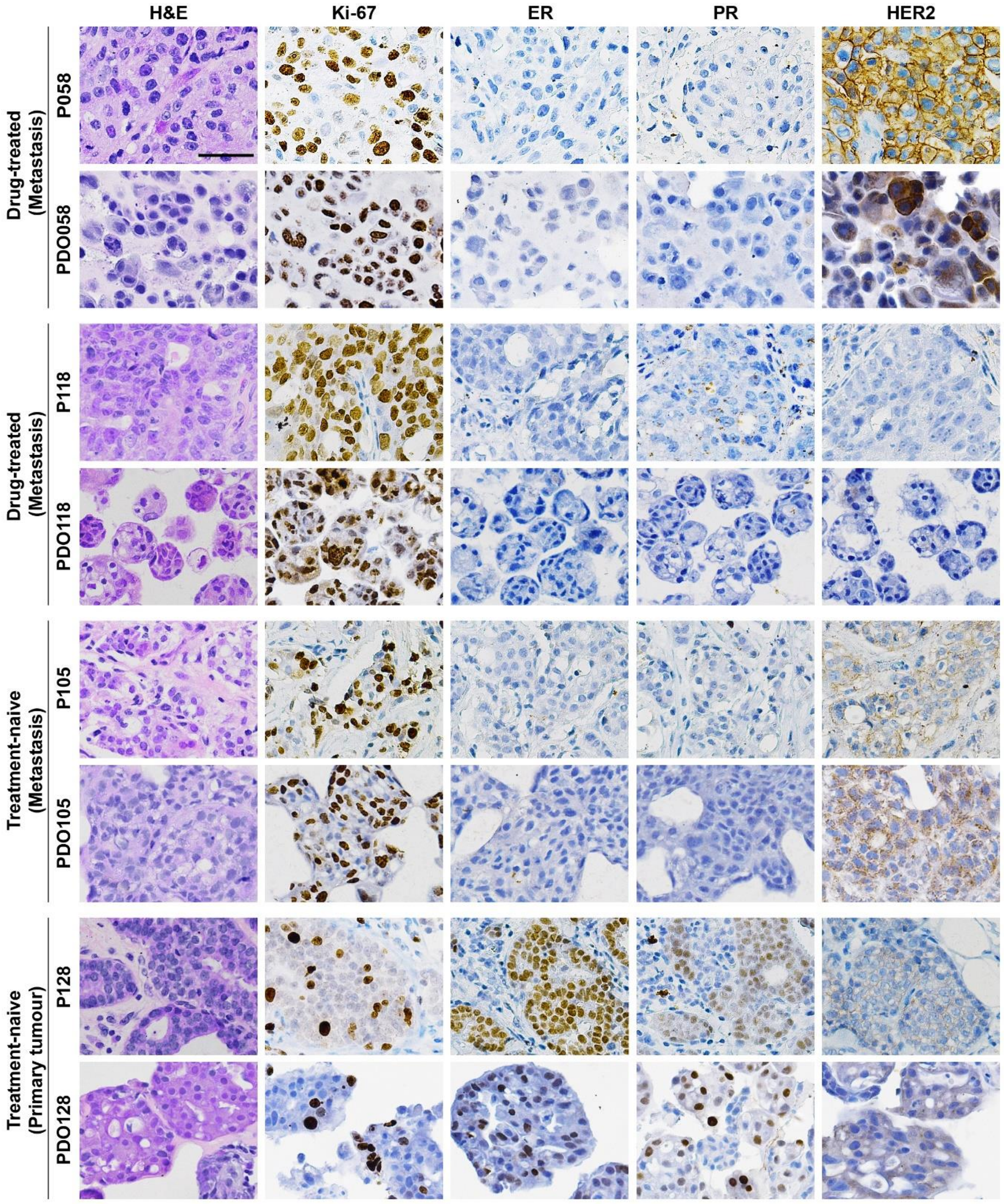

Figure S2. Histological and immunohistochemical analysis of breast cancer organoids and parental tumours.

Histological and immunohistochemical images showing the organization structure and status of proliferation marker (Ki-67) and breast cancer-related markers (ER, PR and HER2) in primary tumours and organoid lines. Scale bar, 50 μm.

Figure S3.

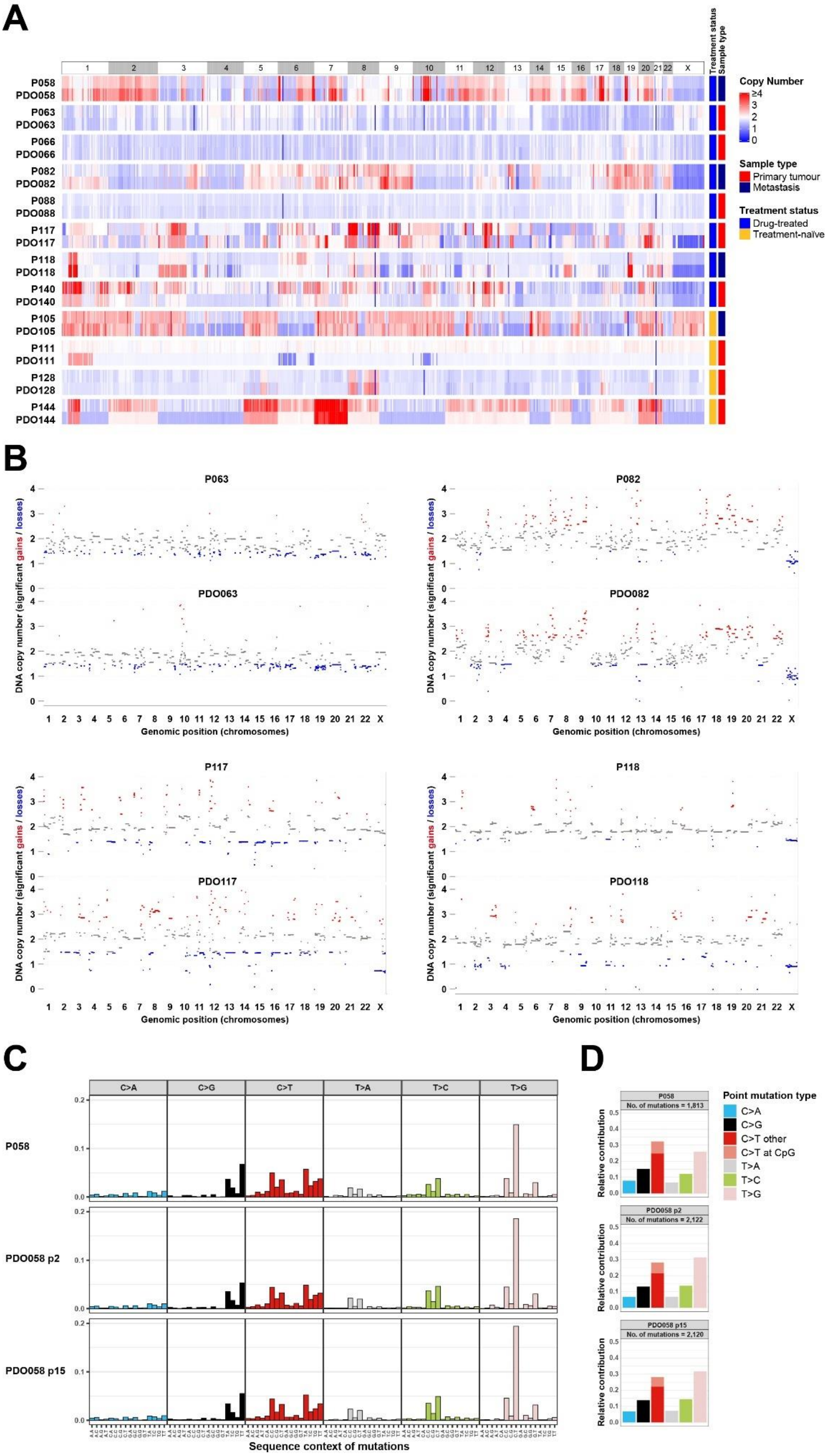
A
Treatment status
Sample type
Copy Number
≥4
3
2
1
0
Sample type
Primary tumour
Metastasis
Treatment status
Drug-treated
Treatment-naïve
P058
PDO058
P063
PDO063
P066
PDO066
P082
PDO082
P088
PDO088
P117
PDO117
P118
PDO118
P140
PDO140
P105
PDO105
P111
PDO111
P128
PDO128
P144
PDO144
B
P063
PDO063
P082
PDO082
P117
PDO117
P118
PDO118
DNA copy number (significant gains / losses)
Genomic position (chromosomes)
C
C>A
C>G
C>T
T>A
T>C
T>G
P058
PDO058 p2
PDO058 p15
Sequence context of mutations
D
Point mutation type
C>A
C>G
C>T other
C>T at CpG
T>A
T>C
T>G
P058
No. of mutations = 1,813
PDO058 p2
No. of mutations = 2,122
PDO058 p15
No. of mutations = 2,120
Relative contribution

Figure S3. Comparison of genomic landscape between breast cancer organoids and parental tumours.

(A) Comparison of the somatic copy number alteration landscape in breast cancer organoids and the parental tumours. The copy number application is shaded in red and the copy number deletion is shaded in blue.

(B) Scatterplots showing genome-wide gene copy number alterations (CNAs) of breast cancer organoids and the parental tumours (red, gains; blue, losses).

(C) Different contributions of point mutation types of primary tumor P058 and the derived organoid line at passage 2 and passage 15 in their sequence context.

(D) Relative contributions of point mutations of primary tumor P058 and the derived organoid line at passage 2 and passage 15.

Figure S4.

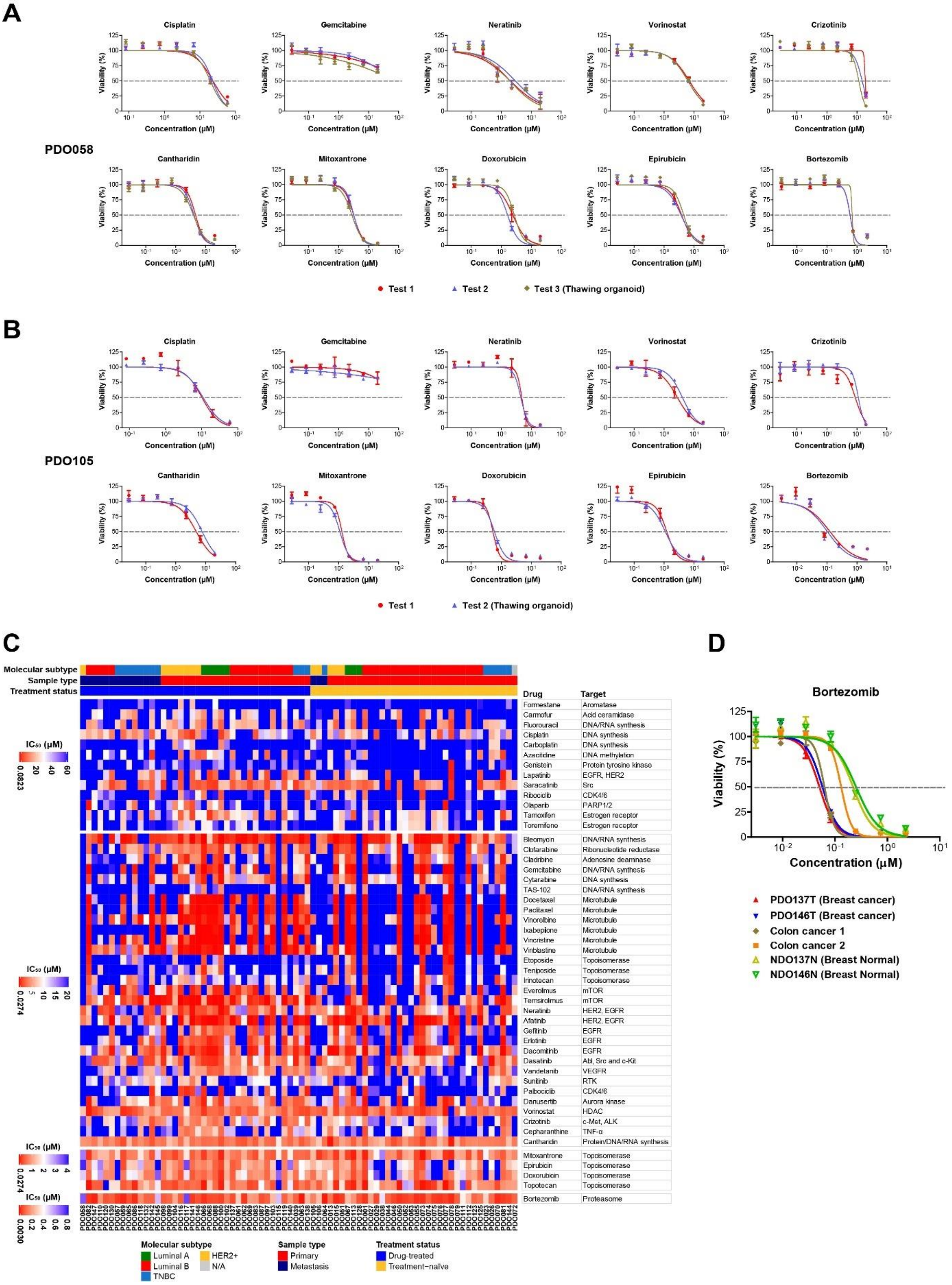
A
PDO058
Cisplatin
Gemcitabine
Neratinib
Vorinostat
Crizotinib
Cantharidin
Mitoxantrone
Doxorubicin
Epirubicin
Bortezomib
Viability (%)
Concentration (μM)
Test 1
Test 2
Test 3 (Thawing organoid)
B
PDO105
Test 1
Test 2 (Thawing organoid)
C
Molecular subtype
Sample type
Treatment status
IC₅₀ (μM)
Drug
Target
Formestane
Aromatase
Carmofur
Acid ceramidase
Fluorouracil
DNA/RNA synthesis
Cisplatin
DNA synthesis
Carboplatin
DNA synthesis
Azacitidine
DNA methylation
Genistein
Protein tyrosine kinase
Lapatinib
EGFR, HER2
Saracatinib
Src
Ribociclib
CDK4/6
Olaparib
PARP1/2
Tamoxifen
Estrogen receptor
Toremifene
Estrogen receptor
Bleomycin
DNA/RNA synthesis
Clofarabine
Ribonucleotide reductase
Cladribine
Adenosine deaminase
Gemcitabine
DNA/RNA synthesis
Cytarabine
DNA synthesis
TAS-102
DNA/RNA synthesis
Docetaxel
Microtubule
Paclitaxel
Microtubule
Vinorelbine
Microtubule
Ixabepilone
Microtubule
Vincristine
Microtubule
Vinblastine
Microtubule
Etoposide
Topoisomerase
Teniposide
Topoisomerase
Irinotecan
Topoisomerase
Everolimus
mTOR
Temsirolimus
mTOR
Neratinib
HER2, EGFR
Afatinib
HER2, EGFR
Gefitinib
EGFR
Erlotinib
EGFR
Dacomitinib
EGFR
Dasatinib
Abl, Src and c-Kit
Vandetanib
VEGFR
Sunitinib
RTK
Palbociclib
CDK4/6
Danusertib
Aurora kinase
Vorinostat
HDAC
Crizotinib
c-Met, ALK
Cepharanthine
TNF-α
Cantharidin
Protein/DNA/RNA synthesis
Mitoxantrone
Topoisomerase
Epirubicin
Topoisomerase
Doxorubicin
Topoisomerase
Topotecan
Topoisomerase
Bortezomib
Proteasome
Molecular subtype
Luminal A
HER2+
Luminal B
N/A
TNBC
Sample type
Primary
Metastasis
Treatment status
Drug-treated
Treatment−naïve
D
Bortezomib
Viability (%)
Concentration (μM)
PDO137T (Breast cancer)
PDO146T (Breast cancer)
Colon cancer 1
Colon cancer 2
NDO137N (Breast Normal)
NDO146N (Breast Normal)

Figure S4. Drug response analyses of breast cancer organoids.

(A) Drug response curves of PDO058 in triplicate testing. The results are expressed as the mean ± SEM.

(B) Drug response curves of PDO105 in two replicated testing. The results are expressed as the mean ± SEM.

(C) Heatmap showing the IC_{50} values of 49 compounds in 76 organoid lines. They were divided into four groups according to the tested range of drug concentrations or IC_{50} values. Does-response graphs of each group are indicated on the left. The tested drugs and their targets are listed on the right. The molecular subtype, sample type and treatment status of the corresponding primary tumour are shown in the top graph.

(D) Drug response curves of breast cancer organoids, colon cancer organoids and breast normal organoids treated with bortezomib. The results are expressed as the mean ± SEM.

Figure S5.

A

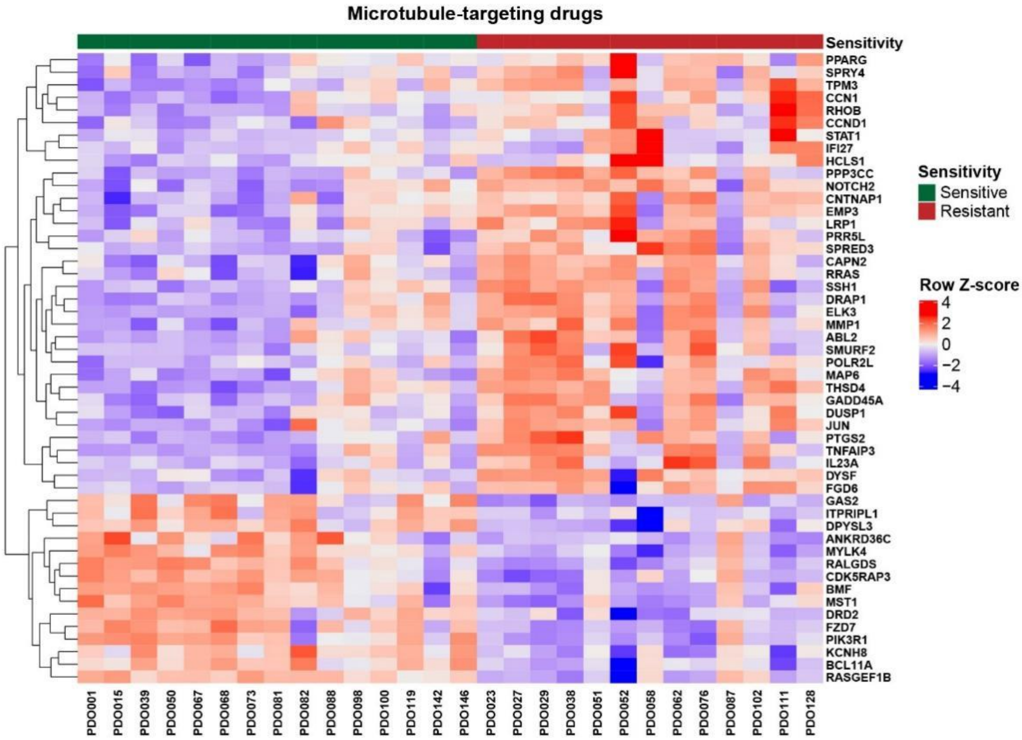

B

	Sensitive_mean	Resistant_mean	P-Value
PPARG	75.45	695.38	8.26882E-05
SPRY4	853.14	3267.92	0.000172811
TPM3	11051.01	20643.74	3.80914E-05
CCN1	1026.82	6868.72	3.49673E-05
RHOB	2178.23	7934.82	8.88865E-06
CCND1	12811.82	32006.74	0.000485992
STAT1	3440.93	9064.57	7.31768E-05
IFI27	71.96	2671.29	5.20427E-05
HCLS1	12.18	144.72	5.89376E-05
PPP3CC	341.48	657.46	3.65761E-06
NOTCH2	5321.96	9534.95	0.000207297
CNTNAP1	272.19	857.40	0.000482837
EMP3	554.62	2664.71	0.000119393
LRP1	6903.99	17229.60	0.000598549
PRR5L	98.87	273.36	1.45599E-05
SPRED3	120.11	445.72	3.53958E-06
CAPN2	15715.85	30332.08	0.000258104
RRAS	2357.89	5178.47	0.000304478
SSH1	2525.44	4664.65	0.000394795
DRAP1	7818.61	21643.74	0.000386021
ELK3	1709.04	4555.13	0.000411138
MMP1	1104.00	37673.04	0.00021678
ABL2	1894.58	3926.17	0.000167649
SMURF2	1556.88	3224.53	1.49583E-05
POLR2L	3358.15	6525.38	0.000134941
MAP6	23.69	150.06	0.0001266
THSD4	1294.02	5656.46	0.000204951
GADD45A	3228.88	7602.92	0.000457675
DUSP1	726.54	2185.31	1.12289E-05
JUN	4145.66	7872.15	5.06889E-05
PTGS2	214.78	3785.64	0.000198927
TNFAIP3	2132.49	12480.63	7.51284E-05
IL23A	33.88	125.17	3.9335E-05
DYSF	893.98	3312.94	9.46689E-05
FGD6	1172.52	2208.15	0.000569301
GAS2	116.04	19.92	0.000171938
ITPRIPL1	610.48	120.16	0.000179515
DPYSL3	1797.86	312.71	0.00050992
ANKRD36C	1824.56	338.26	0.000595631
MYLK4	117.75	37.97	0.000264998
RALGDS	1041.93	354.36	4.87299E-05
CDK5RAP3	1946.06	811.13	0.000448043
BMF	848.74	168.77	0.000205911
MST1	1224.84	363.70	0.000188874
DRD2	83.41	16.33	0.000219468
FZD7	8454.57	1710.66	3.70069E-05
PIK3R1	14060.48	3532.06	9.33415E-05
KCNH8	144.16	19.20	0.000402751
BCL11A	1113.15	318.32	0.000210879
RASGEF1B	594.94	193.36	0.000537878

C

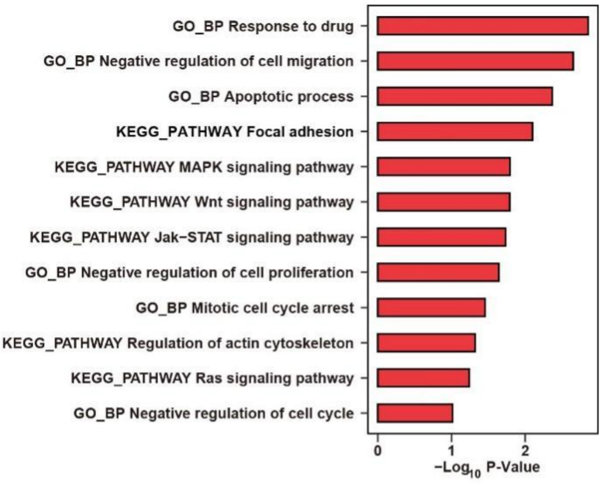

Figure S5. The microtubule-targeting drug-sensitive response signature in breast cancer organoids.

(A) The microtubule-targeting drug-sensitive response signature was generated by comparing different gene expression between the sensitive PDOs (sensitive to all six drugs) and resistant PDOs (resistant to all six drugs). Heat map showing the expression levels of each gene in the PDO lines. Red indicates upregulation of gene expression, while blue indicates downregulation of gene expression.

(B) Summarizing table of the mean expression of each gene in the microtubule-targeting drug sensitive PDOs and resistant PDOs, and p-value between the two groups.

(C) Pathway enrichment analysis identifying functional tendency of the microtubule-targeting drug-sensitive response signature based on GO and the KEGG pathway.

Figure S6.

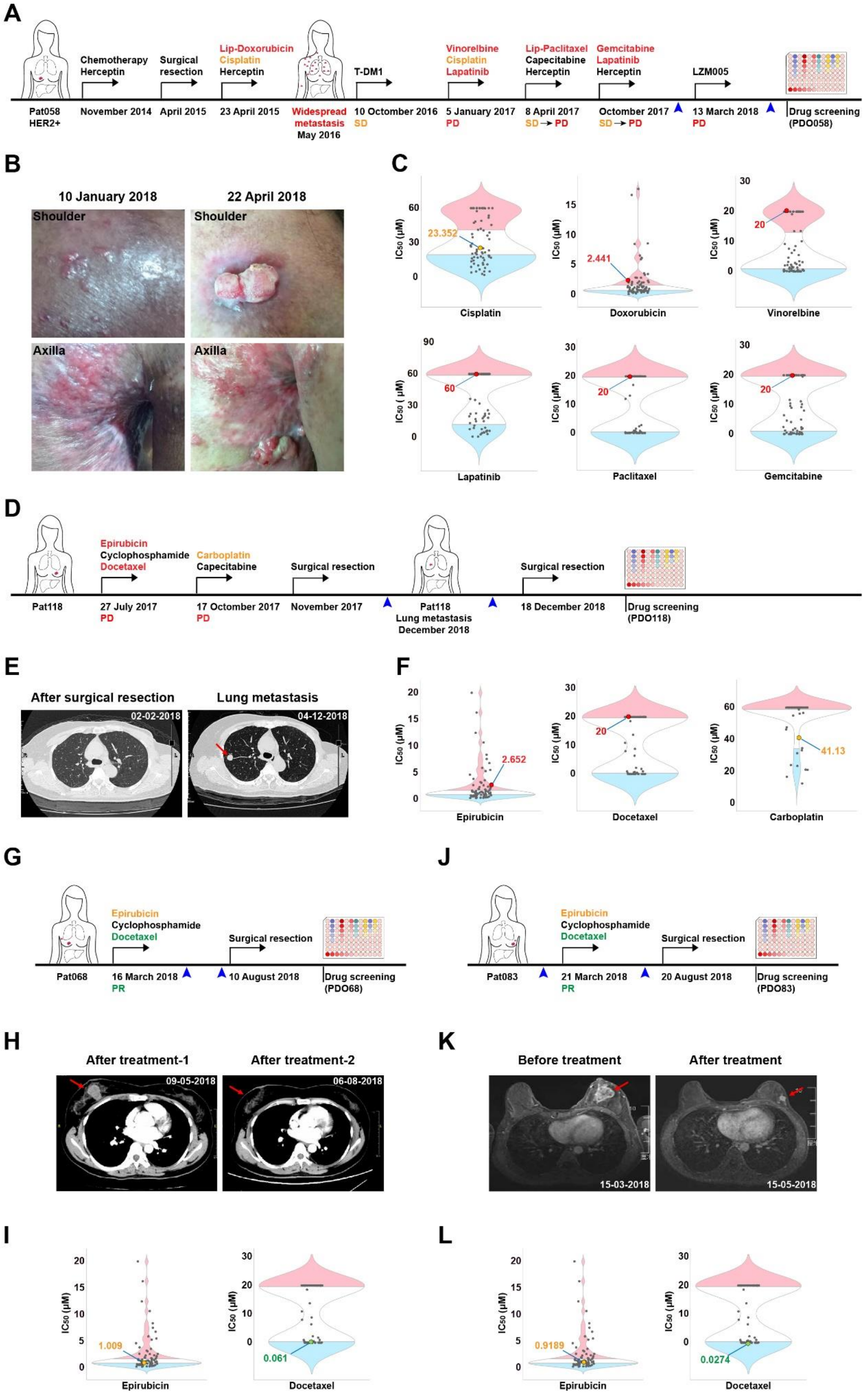
A
Chemotherapy
Herceptin
Surgical resection
Lip-Doxorubicin
Cisplatin
Herceptin
T-DM1
Vinorelbine
Cisplatin
Lapatinib
Lip-Paclitaxel
Capecitabine
Herceptin
Gemcitabine
Lapatinib
Herceptin
LZM005
Pat058
HER2+
November 2014
April 2015
23 April 2015
Widespread metastasis
May 2016
10 Octomber 2016
SD
5 January 2017
PD
8 April 2017
SD → PD
Octomber 2017
SD → PD
13 March 2018
PD
Drug screening
(PDO058)
B
10 January 2018
22 April 2018
Shoulder
Shoulder
Axilla
Axilla
C
IC50 (μM)
23.352
Cisplatin
2.441
Doxorubicin
20
Vinorelbine
60
Lapatinib
20
Paclitaxel
20
Gemcitabine
D
Epirubicin
Cyclophosphamide
Docetaxel
Carboplatin
Capecitabine
Surgical resection
Surgical resection
Pat118
27 July 2017
PD
17 Octomber 2017
PD
November 2017
Pat118
Lung metastasis
December 2018
18 December 2018
Drug screening
(PDO118)
E
After surgical resection
Lung metastasis
02-02-2018
04-12-2018
F
2.652
Epirubicin
20
Docetaxel
41.13
Carboplatin
G
Epirubicin
Cyclophosphamide
Docetaxel
Surgical resection
Pat068
16 March 2018
PR
10 August 2018
Drug screening
(PDO68)
J
Epirubicin
Cyclophosphamide
Docetaxel
Surgical resection
Pat083
21 March 2018
PR
20 August 2018
Drug screening
(PDO83)
H
After treatment-1
After treatment-2
09-05-2018
06-08-2018
K
Before treatment
After treatment
15-03-2018
15-05-2018
I
1.009
Epirubicin
0.061
Docetaxel
L
0.9189
Epirubicin
0.0274
Docetaxel

Figure S6. PDOs retain previous treatment responses of corresponding breast cancer patients.

(A) Treatment procedure and responses of patient Pat058 from surgery till performing drug screening. The therapeutic agents in each round received by the patient are indicated above the arrow and colour coded for *in vitro* drug screening results: intermediate and resistance drug responses are shaded in yellow and red, respectively. Blue arrow tip indicates the images taking time point in panel (B). SD, stable disease. PD, progressive disease.

(B) Tumour images show widespread metastasis of patient Pat058 at different time points.

(C) Violin plot showing the distribution of IC_{50} values of the drugs in the 76 organoid lines, and IC_{50} values of the therapeutic agents received by patient Pat058 are indicated. The blue, white and red parts represent the sensitive, intermediate and resistant samples, respectively.

(D) Treatment procedure and responses of patient Pat118 before performing drug screening. The therapeutic agents received by the patient are indicated above the arrow and colour coded for *in vitro* drug screening results: intermediate and resistance drug responses are shaded in yellow and red, respectively. Blue arrow tips indicate the images taking time points in panel (E). PD, progressive disease.

(E) CT scan images show that patient Pat118 developed lung metastases after treatment. Red arrows indicate tumours.

(F) Violin plot showing the distribution of IC_{50} values of the drugs in the 76 organoid lines, and IC_{50} values of the therapeutic agents received by patient Pat118 are indicated.

(G) Treatment procedure and responses of patient Pat068 before performing drug screening. The therapeutic agents received by the patient are indicated above the arrow and colour coded for *in vitro* drug screening results: intermediate and sensitive drug responses are shaded in yellow and green, respectively. Blue arrow tips indicate the images taking time points in panel (H). PR, Partial response.

(H) Tumour CT scan images show that the tumour size of patient Pat068 was significantly reduced after treatment. Red arrows indicate tumours.

(I) Violin plot showing the distribution of IC_{50} values of the drugs in the 76 organoid lines and IC_{50} values of the therapeutic agents received by patient Pat068 are indicated.

(J) Treatment procedure and responses of patient Pat83 before performing drug screening. The therapeutic agents received by the patient are indicated above the arrow and colour coded for *in vitro* drug screening results: intermediate and sensitive drug responses are shaded in yellow and green, respectively. Blue arrow tips indicate the images taking time points in panel (K). PR, partial response.

(K) Tumour MRI scan images show that the tumour size of patient Pat083 was significantly reduced after treatment. Red arrows indicate tumours.

(L) Violin plot showing the distribution of IC_{50} values of the drugs in the 76 organoid lines, and IC_{50} values of the therapeutic agents received by patient Pat083 are indicated.

Figure S7.

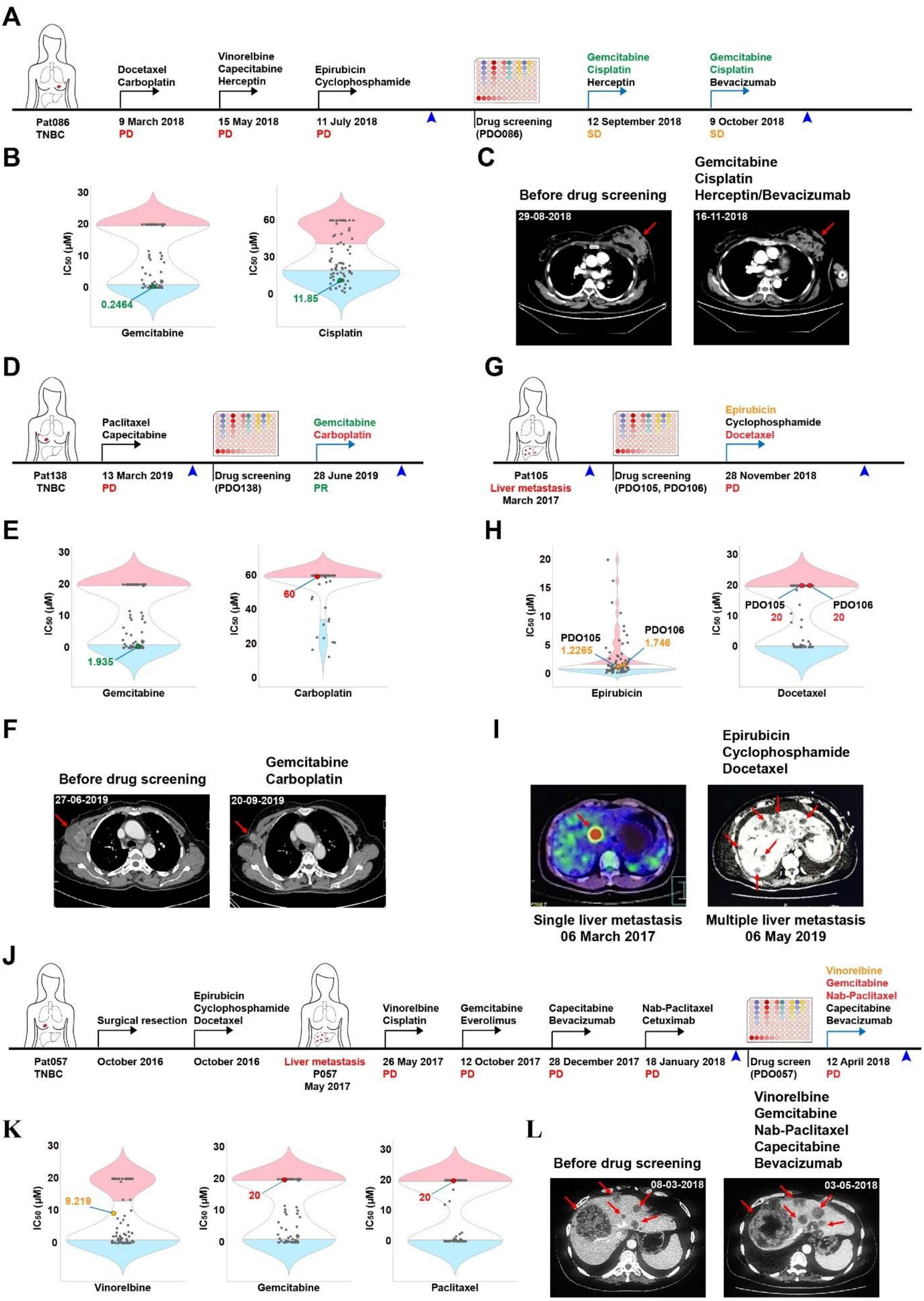

A
Pat086
TNBC
Docetaxel
Carboplatin
9 March 2018
PD
Vinorelbine
Capecitabine
Herceptin
15 May 2018
PD
Epirubicin
Cyclophosphamide
11 July 2018
PD
Drug screening
(PDO086)
Gemcitabine
Cisplatin
Herceptin
12 September 2018
SD
Gemcitabine
Cisplatin
Bevacizumab
9 October 2018
SD
B
IC50 (μM)
0.2464
Gemcitabine
11.85
Cisplatin
C
Before drug screening
29-08-2018
Gemcitabine
Cisplatin
Herceptin/Bevacizumab
16-11-2018
D
Pat138
TNBC
Paclitaxel
Capecitabine
13 March 2019
PD
Drug screening
(PDO138)
Gemcitabine
Carboplatin
28 June 2019
PR
E
1.935
Gemcitabine
60
Carboplatin
F
Before drug screening
27-06-2019
Gemcitabine
Carboplatin
20-09-2019
G
Pat105
Liver metastasis
March 2017
Drug screening
(PDO105, PDO106)
Epirubicin
Cyclophosphamide
Docetaxel
28 November 2018
PD
H
PDO105
1.2265
PDO106
1.746
Epirubicin
PDO105
20
PDO106
20
Docetaxel
I
Epirubicin
Cyclophosphamide
Docetaxel
Single liver metastasis
06 March 2017
Multiple liver metastasis
06 May 2019
J
Pat057
TNBC
Surgical resection
October 2016
Epirubicin
Cyclophosphamide
Docetaxel
October 2016
Liver metastasis
P057
May 2017
Vinorelbine
Cisplatin
26 May 2017
PD
Gemcitabine
Everolimus
12 October 2017
PD
Capecitabine
Bevacizumab
28 December 2017
PD
Nab-Paclitaxel
Cetuximab
18 January 2018
PD
Drug screen
(PDO057)
Vinorelbine
Gemcitabine
Nab-Paclitaxel
Capecitabine
Bevacizumab
12 April 2018
PD
K
9.219
Vinorelbine
20
Gemcitabine
20
Paclitaxel
L
Before drug screening
08-03-2018
Vinorelbine
Gemcitabine
Nab-Paclitaxel
Capecitabine
Bevacizumab
03-05-2018

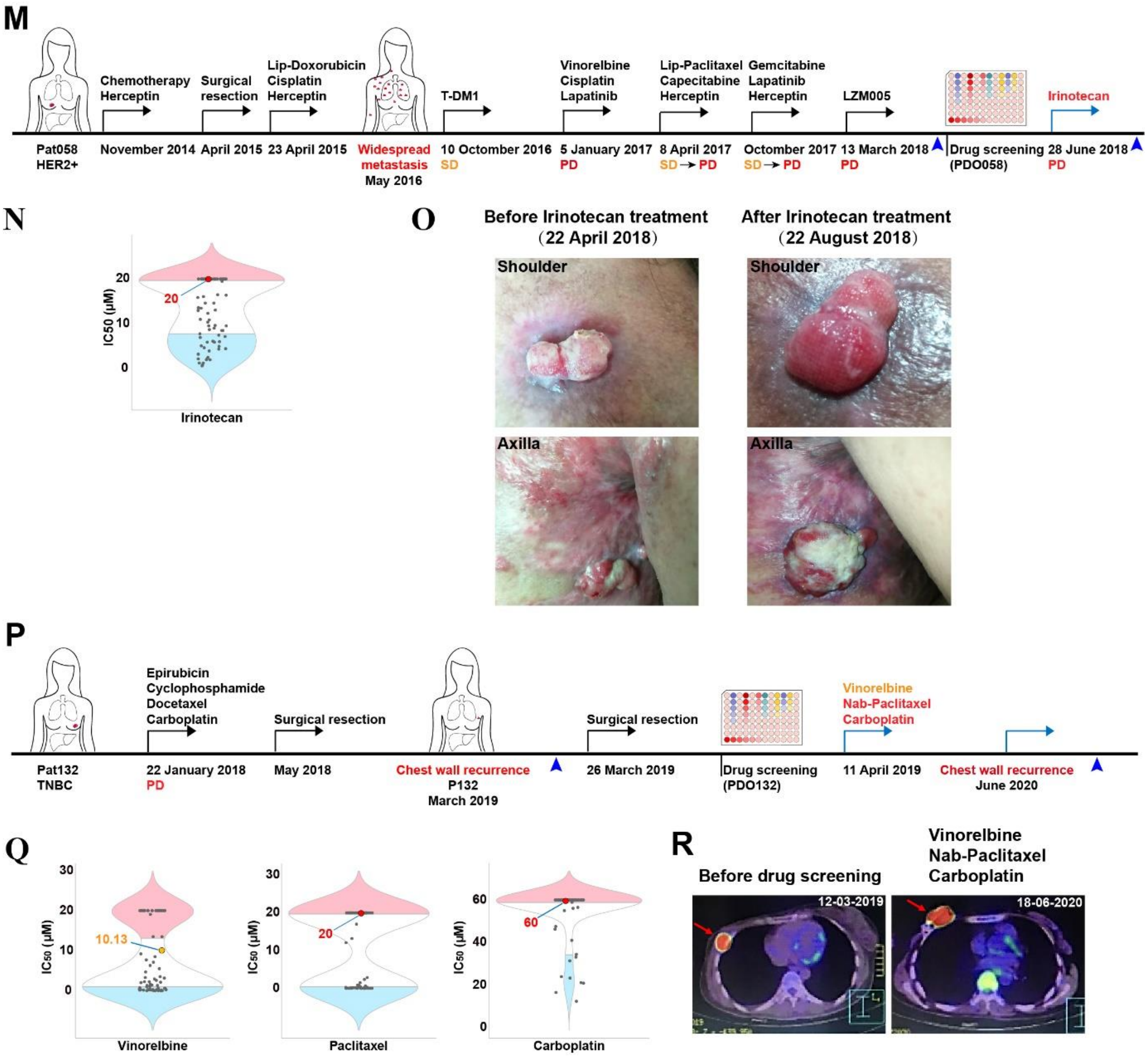

Figure S7. The PDOs platform predicts drug-responses and patients' clinical outcomes.

(A) Treatment procedures and responses of patient Pat086. The therapeutic agents in each round received by the patient are indicated above the arrow. Blue arrow tips indicate the images taking time points in panel (C). PD, progressive disease. SD, stable disease.

(B) Violin plot showing the distribution of IC_{50} values of the drugs in the 76 organoid lines and IC_{50} values of the therapeutic agents received by patient Pat086 after drug screening are indicated. The blue, white and red parts represent the sensitive, intermediate and resistant samples, respectively.

(C) Tumour CT scan images of patient Pat086 before and after personalized therapy (gemcitabine, cisplatin and Herceptin/bevacizumab). Red arrows indicate tumours.

(D) Treatment procedures and responses of patient Pat138. The therapeutic agents in each round received by the patient are indicated above the arrow. Blue arrow tips indicate the images taking time points in panel (F). PD, progressive disease. PR, partial response.

(E) Violin plot showing the distribution of IC_{50} values of the drugs in the 76 organoid lines and IC_{50} values of the therapeutic agents received by patient Pat138 after drug screening are indicated.
(F) Tumour CT scan images of patient Pat138 before and after personalized therapy (gemcitabine and carboplatin). Red arrows indicate tumours.
(G) Treatment procedures and responses of patient Pat105. The therapeutic agents received by the patient are indicated above the arrow. Blue arrow tips indicate the images taking time points in panel (I). PD, progressive disease.
(H) Violin plot showing the distribution of IC_{50} values of the drugs in the 76 organoid lines and IC_{50} values of the therapeutic agents received by patient Pat105 after drug screening are indicated.
(I) Tumour PET-CT and CT scan images of patient Pat105 before and after therapy (epirubicin, cyclophosphamide and docetaxel). Red arrows indicate tumours.
(J) Treatment procedures and responses of patient Pat057. The therapeutic agents received by the patient are indicated above the arrow. Blue arrow tips indicate the images taking time points in panel (L). PD, progressive disease.
(K) Violin plot showing the distribution of IC_{50} values of the drugs in the 76 organoid lines and IC_{50} values of the therapeutic agents received by patient Pat057 after drug screening are indicated.
(L) Tumour CT scan images of patient Pat057 before and after therapy (vinorelbine, gemcitabine, nab-paclitaxel, capecitabine and bevacizumab). Red arrows indicate tumours.
(M) Treatment procedures and responses of patient Pat058. The therapeutic agents received by the patient are indicated above the arrow. Blue arrow tips indicate the images taking time points in panel (O). SD, stable disease. PD, progressive disease.
(N) Violin plot showing the distribution of IC_{50} values of the drugs in the 76 organoid lines and IC_{50} values of the therapeutic agents received by patient Pat058 after drug screening are indicated.
(O) Tumour images of patient Pat058 before and after treatment (irinotecan). The tumour images before irinotecan treatment are also shown in Figure S6B.
(P) Treatment procedures and responses of patient Pat132. The therapeutic agents received by the patient are indicated above the arrow. Blue arrow tips indicate the images taking time points in panel (R). PD, progressive disease.

(Q) Violin plot showing the distribution of IC_{50} values of the drugs in the 76 organoid lines and IC_{50} values of the therapeutic agents received by patient Pat132 after drug screening are indicated.

(R) Tumour CT scan images of patient Pat132 before and after therapy (vinorelbine, nab-paclitaxel and carboplatin). Red arrows indicate tumours.

Figure S8.

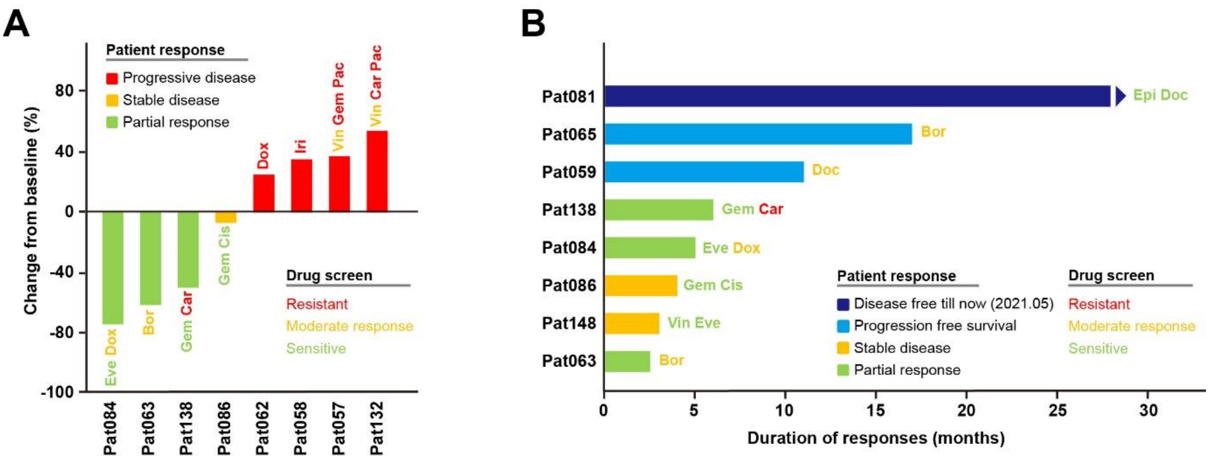

Figure S8. Summary of PDO predicated drug-responses and patients' clinical outcomes in Table 1.

Pat081 received surgical resection after the treatment. Pat065 and Pat059 had metastatic lesions, and the visible lesions had been surgically removed before treatment. The tumour of Pat148 less than 10 mm in diameter and the tumour of Pat105 was evaluated by ultrasonography.

(A) Waterfall plot of the percent tumour response after treatment for patients in Table 1. The responses of PDOs to the drugs are indicated.

(B) The duration of patients' responses to the treatments in Table 1.

Table S1. The number of CNA genes in tumours and organoids.

Patient ID	Tumour	Organoid	Common	Common_Percentage (%)
Pat058	2862	11015	2810	98.18
Pat063	7903	8858	7148	90.45
Pat066	796	1690	618	77.64
Pat082	5111	7947	4463	87.32
Pat088	6	192	2	33.33
Pat117	7036	8762	3821	54.31
Pat118	2275	5909	1775	78.02
Pat140	9338	2632	2438	26.10
Pat105	9889	11948	5527	55.89
Pat111	172	1582	17	9.88
Pat128	542	1608	526	97.05
Pat144	8931	10446	5088	56.97

Table S2. The composition of the organoid culture medium.

Additive	Supplier	Cat. No.	Concentration
Advanced DMEM/F12	GIBCO	Cat# 12634-010	Base medium
Wnt3A	Conditional medium	N/A	30% v/v
R-spondin 1	Conditional medium	N/A	10% v/v
Noggin	Conditional medium	N/A	10% v/v
Y-27632	DC Chemicals	Cat# DC1028	5 μM
SB202190	DC Chemicals	Cat# DC2097	0.5 μM
A83-01	DC Chemicals	Cat# DC7286	0.5 μM
EGF	PeproTech	Cat# AF-100-15	5 ng/ml
Neuregulin-1	PeproTech	Cat# 100-03	5 nM
Hydrocortisone	Sigma-Aldrich	Cat# H0888-10G	500 ng/ml
N-Acetyl-L-cysteine	Sigma-Aldrich	Cat# A9165-100G	1.25 mM
HEPES	Sigma-Aldrich	Cat# H3375-1KG	15 mM
B27 Supplement (50X)	GIBCO	Cat# 17504-001	1×
Glutamax	GIBCO	Cat# 35050-061	1×
β-Estradiol	Sigma-Aldrich	Cat# E2758-5G	5 nM
Insulin-Transferrin-Selenium-Sodium Pyruvate (ITS-A)	GIBCO	Cat# 51300044	1×
Amphotericin B	GIBCO	Cat# 15290-018	0.5 μg/ml
Gentamicin	GIBCO	Cat# 15710-064	5 μg/ml
Plasmocin	InvivoGen	Cat# ANT-MPP	5 μg/ml

Table S3. Cell lines used in this study.

Cell lines	Supplier	Identifier
L Wnt-3A cell line	ATCC	Cat# CRL-2647
R-spondin 1 T-REx-293 cell line	This study	N/A
Noggin T-REx-293 cell line	This study	N/A
T-REx-293 cell line	ThermoFisher	Cat# R71007

Table S4. Antibodies used in immunohistochemistry and western blotting assays.

Antibodies	Supplier	Identifier
Ki-67 (8D5) Mouse monoclonal antibody	Cell Signaling Technology	Cat# 9449S; RRID: AB_2797703
Recombinant Anti-Estrogen Receptor alpha antibody [SP1]	Abcam	Cat# ab16660; RRID: AB_443420
Recombinant Anti-Progesterone Receptor antibody [SP42]	Abcam	Cat# ab101688; RRID: AB_10715248
HER2/ErbB2 (29D8) Rabbit monoclonal antibody	Cell Signaling Technology	Cat# 2165; RRID: AB_10692490
Phospho-Stat3 (Tyr705) (D3A7) XP Rabbit monoclonal antibody	Cell Signaling Technology	Cat# 9145S; RRID: AB_2491009
Stat3 (124H6) Mouse monoclonal antibody	Cell Signaling Technology	Cat# 9139S; RRID: AB_331757
cyclin A (H-432) Rabbit polyclonal antibody	SANTA CRUZ BIOTECHNOLOGY	Cat# sc-751; RRID: AB_631329
cyclin B1 (H-433) Rabbit polyclonal antibody	SANTA CRUZ BIOTECHNOLOGY	Cat# sc-752; RRID: AB_2072134
Phospho-Bcl-2 (Ser70) (5H2) Rabbit monoclonal antibody	Cell Signaling Technology	Cat# 2827S; RRID: AB_659950
Bcl-2 Rabbit polyclonal antibody	Proteintech	Cat# 12789-1-AP; RRID: AB_2227948
Cleaved Caspase-3 (Asp175) Rabbit antibody	Cell Signaling Technology	Cat# 9661S; RRID: AB_2341188
Monoclonal Anti-β-Actin antibody produced in mouse	Sigma-Aldrich	Cat# A5316; RRID: AB_476743
Anti-rabbit IgG, HRP-linked antibody	Cell Signaling Technology	Cat# 7074S; RRID: AB_2099233
Anti-mouse IgG, HRP-linked antibody	Cell Signaling Technology	Cat# 7076S; RRID: AB_330924

Table S5. Drugs tested in this study.

Drugs	Supplier	Cat. No.
Formestane	Selleckchem	Cat# S2208
Carmofur	Selleckchem	Cat# S1289
Fluorouracil	Selleckchem	Cat# S1209
Cisplatin	Sigma-Aldrich	Cat# P4394-1G
Carboplatin	Sigma-Aldrich	Cat# C2538-250MG
Azacitidine	Selleckchem	Cat# S1782
Genistein	Selleckchem	Cat# S1342
Lapatinib	Selleckchem	Cat# S1028
Saracatinib	Selleckchem	Cat# S1006
Ribociclib	Selleckchem	Cat# S7440
Olaparib	Selleckchem	Cat# S1060
Tamoxifen	Selleckchem	Cat# S1972
Toremifene	Selleckchem	Cat# S1776
Bleomycin	Selleckchem	Cat# S1214
Clofarabine	Selleckchem	Cat# S1218
Cladribine	Selleckchem	Cat# S1199
Gemcitabine	Selleckchem	Cat# S1149
Cytarabine	Selleckchem	Cat# S1648
TAS-102	Selleckchem	Cat# S8539
Docetaxel	Selleckchem	Cat# S1148
Paclitaxel	Selleckchem	Cat# S1150
Vinorelbine	Selleckchem	Cat# S4269
Ixabepilone	Selleckchem	Cat# S7930
Vincristine	Selleckchem	Cat# S1241
Vinblastine	Selleckchem	Cat# S4505
Etoposide	Selleckchem	Cat# S1225
Teniposide	J&K Scientific	Cat# 563738
Irinotecan	Selleckchem	Cat# S1198
Everolimus	Selleckchem	Cat# S1120
Temsirolimus	Selleckchem	Cat# S1044
Neratinib	Selleckchem	Cat# S2150
Afatinib	Selleckchem	Cat# S1011
Gefitinib	Selleckchem	Cat# S1025
Erlotinib	Selleckchem	Cat# S1023
Dacomitinib	Selleckchem	Cat# S2727
Dasatinib	Selleckchem	Cat# S1021
Vandetanib	Selleckchem	Cat# S1046
Sunitinib	Selleckchem	Cat# S1042
Palbociclib	Selleckchem	Cat# S1116
Danusertib	Selleckchem	Cat# S1107
Vorinostat	Selleckchem	Cat# S1047
Crizotinib	Selleckchem	Cat# S1068
Cepharanthine	Selleckchem	Cat# S4238
Cantharidin	Sigma-Aldrich	Cat# C7632
Mitoxantrone	Selleckchem	Cat# S2485
Epirubicin	J&K Scientific	Cat# 194237
Doxorubicin	Selleckchem	Cat# S1208
Topotecan	Selleckchem	Cat# S1231
Bortezomib	BOC Sciences	Cat# B0084-293315

Table S6. Software used in this study.

Software	Reference	Download link
BWA-MEM (v0.7.17)	Li and Durbin, 2009	https://github.com/lh3/bwa
MultiQC (v1.7)	Ewels et al., 2016	https://multiqc.info/
SAMtools (v0.1.9)	Li, 2011; Li et al., 2009	https://github.com/samtools/samtools
Genome Analysis ToolKit (v4.1.1.0)	DePristo et al., 2011; McKenna et al., 2010; Van der Auwera et al., 2013	https://github.com/broadinstitute/gatk
MuTect (v1.1.7)	Cibulskis et al., 2013	https://github.com/broadinstitute/mutect
Strelka (v2.9.10)	Saunders et al., 2012	https://github.com/Illumina/strelka
Annovar (v2018-04-16)	Wang et al., 2010	https://doc-openbio.readthedocs.io/projects/annovar/en/latest/
Control-FREEC (v11.4)	Boeva et al., 2012	http://boevalab.inf.ethz.ch/FREEC/
R package MutationalPatterns (v1.10.0)	Blokzijl et al., 2018	https://bioconductor.org/packages/release/bioc/html/MutationalPatterns.html
STAR (v2.7.0e)	Dobin et al., 2013	https://github.com/alexdobin/STAR
featureCounts (v1.6.4)	Liao et al., 2014	http://bioinf.wehi.edu.au/featureCounts/
edgeR package (v3.26.8)	Robinson et al., 2010	http://bioconductor.org/packages/release/bioc/html/edgeR.html
R package ‘e1071’ (v1.7-3)	David et al., 2019	https://CRAN.R-project.org/package=e1071
